# Virgin coconut oil: A comprehensive review of its health impacts and functional food applications

**DOI:** 10.1016/j.fochx.2026.104002

**Published:** 2026-05-20

**Authors:** Imad Khan, Zehua Qiu, Jianguo Zhang, Rui Li, Zhiyou Yang, Weimin Zhang, Shucheng Liu, Minmin Tang, Qiuyu Xia

**Affiliations:** a College of Food Science and Technology, Guangdong Ocean University, Guangdong Provincial Key Laboratory of Aquatic Product Processing and Safety, Guangdong Province Engineering Laboratory for Marine Biological Products, Guangdong Provincial Engineering Technology Research Center of Seafood, Guangdong Provincial Engineering Technology Research Center of Prefabricated Seafood Processing and Quality Control, Zhanjiang 524088, China; b Coconut Research Institute, Chinese Academy of Tropical Agricultural Sciences, Wenchang, Hainan 571339, China; c College of Food Science and Engineering, Key Laboratory of Food Nutrition and Functional Food of Hainan Province, Hainan University, Haikou 570228, China

**Keywords:** Virgin coconut oil, Health benefits, Functional food applications

## Abstract

Virgin coconut oil (VCO) has gained attention as a functional food ingredient due to its unique fatty acid profile, particularly medium-chain fatty acids (MCFAs), and minor bioactive compounds. This review synthesizes recent preclinical and clinical evidence on the biological activities, safety, and functional food applications of VCO. Experimental studies report antioxidant, anti-inflammatory, antimicrobial, metabolic, and skin-protective effects, mediated through modulation of oxidative stress, inflammatory signaling pathways (including NF-κB and Nrf2), lipid metabolism, and cellular barrier functions. Human studies suggest potential benefits related to antioxidant status, inflammatory modulation, metabolic regulation, and skin health, although outcomes vary with study design and intake levels. Technologically, VCO shows strong potential in food systems by enhancing oxidative stability, texture, and bioactive delivery in oleogels, emulsions, and encapsulated formulations. Overall, this review integrates mechanistic and applied evidence, highlighting VCO as a multifunctional ingredient while emphasizing the need for robust clinical trials and optimized delivery strategies.

## Highlights

• Overview of VCO bioactive composition and key biological mechanisms.
• Evidence of antioxidant, anti-inflammatory, metabolic, and antimicrobial effects.
• Summary of recent preclinical and human studies on VCO functionality.
• Applications of VCO in functional foods, oleogels, emulsions, and delivery systems.
• Future prospects for VCO-based food and nutraceutical innovations.

## Graphical abstract

**Figure:**
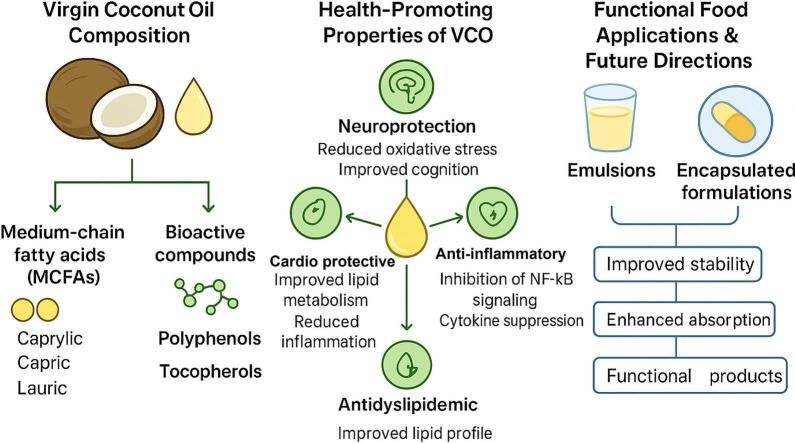

## 1 Introduction

Coconut (*Cocos nucifera L*.), a member of the Arecaceae (palm) family, is widely cultivated across tropical and subtropical regions. The coconut fruit consists of approximately 51.7% (w/w) kernel, 9.8% (w/w) water, and 38.5% (w/w) shell (Divya et al., 2023). The kernel is particularly rich in essential minerals, including calcium, phosphorus, iron, magnesium, and selenium, as well as vitamins E, C, B3, B1, B5, and B6 (Henrietta et al., 2022). These nutrient-dense characteristics have contributed to the growing global demand for value-added coconut-derived products, driven by increased consumer interest in functional and health-promoting foods. Among coconut-based products, coconut oil has gained popularity, particularly in Western markets, owing to its distinctive nutritional composition and versatility in food applications. According to the Asian Pacific Coconut Community (APCC), coconut oil exports from Asia exhibited an average annual growth rate of 3.3% over a five-year period, reflecting expanding international demand (Athauda et al., 2015).

Virgin coconut oil (VCO) is obtained from fresh coconut kernel through minimal processing without high-temperature treatment or chemical refining, thereby preserving its naturally occurring nutrients and bioactive compounds (Rohman & Indrayanto, 2024). The nutritional and functional significance of VCO is largely attributed to its unique fatty acid profile, which is dominated by MCFAs), particularly lauric acid (48-53%) (Agarwal & Bosco, 2017), along with other biologically active MCFAs (Dias et al., 2018). Owing to this composition, VCO is widely recognized as a functional dietary lipid. Lauric acid plays a central role among these fatty acids, as it is metabolized into monolaurin, a compound reported to support immune function and exhibit antibacterial and antiviral activity, especially in early life stages (Nitbani et al., 2022).

In addition to MCFAs, VCO contains a diverse range of secondary phytochemicals, including tocopherols, polyphenols, flavonoids, tocotrienols, vitamin A, and phytosterols. These constituents are closely associated with antioxidant and anti-inflammatory activities and contribute to the biological functionality of VCO (Angeles-Agdeppa et al., 2021; Kumari & Jamwal, 2023). A growing body of experimental and clinical research has explored the health-related properties of VCO, reporting antioxidant, anti-inflammatory, neuroprotective, metabolic, cardiovascular, and antimicrobial effects (Demirel et al., 2024; Nasution et al., 2023; Rahman et al., 2024; Sinaga et al., 2020). These effects are attributed to the synergistic actions of MCFAs and phytochemical components, which collectively support metabolic regulation and physiological resilience (Bafail et al., 2024).

The aim of this review is to critically synthesize recent evidence on the health effects, safety considerations, and functional food applications of VCO. By systematically evaluating preclinical and human studies published over the past five years, this article examines the antioxidant, anti-inflammatory, cardiometabolic, antimicrobial, neuroprotective, and tissue-protective properties of VCO, with particular emphasis on mechanistic pathways and translational relevance. In addition, the review discusses dosage considerations, potential limitations, and inconsistencies in the existing literature, while highlighting emerging technological strategies for incorporating VCO into functional foods and nutraceutical formulations. By integrating biological mechanisms with practical applications, this work aims to clarify the current scientific understanding of VCO and identify key research gaps to guide future clinical investigations and industrial development.

## 2 Chemical composition of VCO

VCO is recognized for its unique chemical composition, primarily characterized by a high content of MCFAs and various bioactive compounds, which collectively contribute to its functional properties in food systems, including oxidative stability and emulsification capacity (Zeng et al., 2024; Zainodin et al., 2024; Prasanna et al., 2024). The extraction of VCO from fresh coconut meat involves minimal processing, often without the use of harsh chemicals or high heat, preserving its beneficial constituents (Zeng et al., 2024; Prasanna et al., 2024). Different processing methods, such as cold pressing, centrifugation, enzymatic extraction, and fermentation, can influence the yield and quality of VCO, affecting the final concentration of these key compounds (Ngampeerapong et al., 2018; Nivya et al., 2023; Sutanto et al., 2021).

The predominant fatty acids in VCO are saturated, with approximately 90% saturated fatty acid content (Sabahannur & Alimuddin, 2022). Lauric acid (C12:0) is the most abundant MCFA, typically ranging from 45% to 52% (Nitbani et al., 2022). Other significant MCFAs include caprylic acid (C8:0) and capric acid (C10:0), alongside myristic acid (C14:0) (Mandey et al., 2025). For instance, a study identified lauric acid as the highest component in VCO at 45.567% (Sabahannur & Alimuddin, 2022). These MCFAs are distinct from long-chain fatty acids because of their shorter carbon tails, which grant them unique metabolic pathways and physicochemical properties (Duranova et al., 2025). This includes easier digestion and absorption into the portal vein, bypassing the lymphatic system, and rapid mitochondrial entry for energy production without the carnitine shuttle system (Duranova et al., 2025). The saturation and shorter chain length of MCFAs inherently contribute to VCO's high oxidative stability.

Moreover, beyond fatty acids, VCO contains an array of minor bioactive components that enhance its functional attributes. These include polyphenols, tocopherols (vitamin E), phytosterols, and squalene (Lantemona, 2023). Polyphenols, such as gallic acid, caffeic acid, and p-coumaric acid (P-CA), are potent antioxidants that play a crucial role in preventing lipid oxidation in VCO (Lantemona, 2023). Tocopherols, another class of antioxidants, also contribute to oxidative stability by scavenging free radicals (Zainodin et al., 2024). The combined action of these antioxidants helps extend the shelf life of VCO and preserves its nutritional quality (Zeng et al., 2024). Studies have shown that VCO exhibits superior oxidative stability compared to refined coconut oil, demonstrating lower free fatty acid content and peroxide values over time under accelerated aging conditions.

The chemical composition of VCO directly influences its functional behavior in food systems, particularly its oxidative stability and emulsification properties. Oxidative Stability: The high proportion of saturated MCFAs in VCO makes it inherently resistant to oxidation, a primary cause of spoilage in oils. Saturated fatty acids lack double bonds, which are the primary sites for radical attack during lipid peroxidation (Van Nguyen & Shahidi, 2025). This structural characteristic, coupled with the presence of natural antioxidants like polyphenols and tocopherols, significantly enhances VCO's resistance to rancidity (Zainodin et al., 2024). The synergism between MCFAs and these antioxidant compounds helps to inhibit the formation of hydroperoxides, which are key indicators of oxidative degradation. Incorporating natural antioxidants from sources like strawberry, cinnamon, beetroot, and ginger can further extend VCO's shelf life (Rangani & Ranaweera, 2023). VCO extracted using methods like microwave heating and ultrasonication have been found to retain its beneficial antioxidants, contributing to its nutraceutical potential.

Emulsification is a critical property for incorporating oils into various food products, cosmetic lotions, and drug delivery systems (Jose et al., 2022). VCO's unique fatty acid profile, including the relatively short chain length of its dominant MCFAs, and its low viscosity, facilitate easier dispersion and interfacial film formation when combined with emulsifiers (Xiao et al., 2022). MCFAs contribute to the rapid formation of micelles and exhibit interfacial activity, enhancing emulsification capacity, particularly in systems with varying pH or salt concentrations. The emulsification capacity of VCO can be crucial in developing products like oil-in-water emulsions, microencapsulated powders, and lotions (Kulandasamy et al., 2023). For instance, a study on noni fruit extract emulsions in VCO explored the effect of different surfactants (Cremophor EL, Cremophor RH 40, and Tween 80) on emulsion stability (Jose et al., 2022). The stability of VCO emulsions is vital for maintaining product quality and extending shelf life, with research showing stable VCO emulsions for up to 12 months under room temperature storage (Wiyani et al., 2023). Microencapsulation techniques, such as O/W emulsification and complex coacervation, are employed to convert liquid VCO into a more consumer-friendly powder form, leveraging its emulsification properties for effective encapsulation and controlled release of its beneficial compounds (Kulandasamy et al., 2023). The use of VCO in film-forming emulsions, incorporating corn starch, sodium alginate, and gum Arabic, has shown that increased VCO content can affect emulsion stability and film properties, such as water vapor permeability (Xiao et al., 2022).

In summary, the high concentration of saturated MCFAs, particularly lauric acid, combined with the presence of natural antioxidants like polyphenols and tocopherols, supports VCO's excellent oxidative stability and favorable emulsification characteristics. These properties make VCO a valuable ingredient in a wide range of applications, including functional foods, pharmaceuticals, and cosmetics. The ongoing research into VCO's chemical profile and functional attributes continues to highlight its importance and versatility.

## 3 Comparison of VCO with other functional oils

The comparative evaluation of VCO against selected functional oils is essential for clarifying their distinctive nutritional and metabolic characteristics within the broader context of dietary lipids. Comparing VCO with olive and palm oils provides insights into how its unique fatty acid composition translates into specific physiological advantages and practical applications in food and industrial settings (Rabail et al., 2021).

VCO is distinguished by its exceptionally high content of MCFAs, with lauric acid (C12:0) comprising approximately 45-50% of total fatty acids, followed by myristic acid (C14:0, 16-20%) and caprylic acid (C8:0, 6-8%) (Srivastava et al., 2018; Wallace, 2019). These MCFAs are rapidly absorbed through the portal circulation and preferentially oxidized for energy rather than stored as adipose tissue, providing metabolic advantages. Additionally, VCO demonstrates remarkable oxidative stability due to its saturated MCFA content, making it suitable for high-temperature cooking and industrial applications. VCO also contains bioactive compounds, including polyphenols, tocopherols, and phytosterols, which enhance their antioxidant capacity and functional properties (Wallace, 2019).

In contrast, extra virgin olive oil (EVOO) is rich in monounsaturated fatty acids (oleic acid, C18:1, 70-77%) and phenolic compounds such as hydroxytyrosol and oleuropein, which contribute to its cardioprotective effects and antioxidant properties (Gorzynik-Debicka et al., 2018; Maslova et al., 2018). While EVOO offers significant metabolic benefits, its oxidative stability is moderate, limiting its use in high-temperature cooking.

Palm oil, with palmitic acid (C16:0, 40-45%) and oleic acid (C18:1, 35-40%), provides semi-solid consistency and good thermal stability for food processing applications (Mancini et al., 2015; Sambanthamurthi et al., 2000). However, concerns remain regarding high saturated fat intake and potential cardiovascular risks.

This comparison highlights VCO’s unique position among functional oils, it combines rapid metabolic utilization of MCFAs, high oxidative stability suitable for industrial applications, and bioactive compounds contributing to functional health benefits. By contrast, olive oil primarily offers cardioprotective advantages, and palm oil provides processing stability but with potential health concerns. Therefore, VCO emerges as a versatile functional oil suitable for both nutritional and industrial purposes (Rabail et al., 2021; Selvaraj et al., 2020).

## 4 Health implications of VCO

**Fig. 1:**
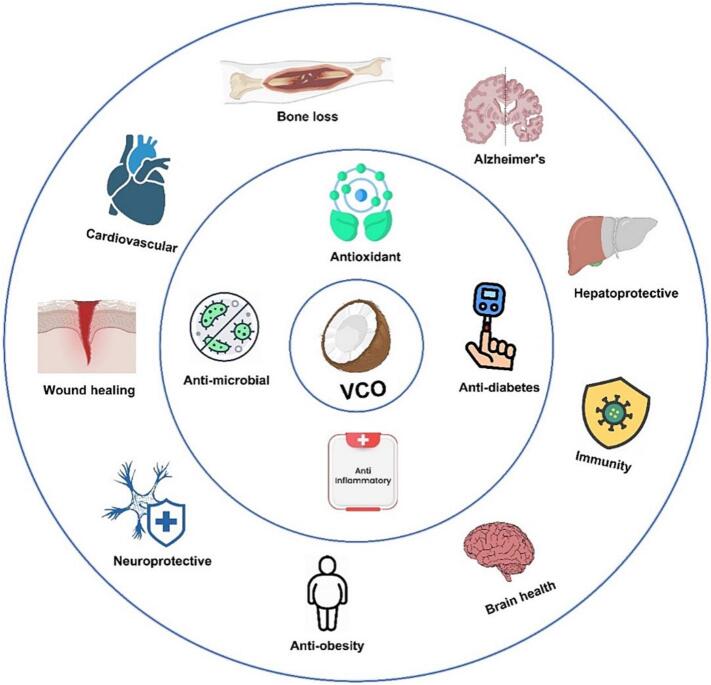
Schematic overview of the potential health effects associated with VCO. Most findings are based on preclinical or short-term human studies, so these effects should be interpreted with caution due to limited long-term clinical evidence.

VCO is referred to as "raw coconut oil" which is widely known for its medicinal usages. In the Philippines it is referred to as the "drugstore in a bottle" due to its therapeutic properties. Additionally, it has been used for healing purposes in India for centuries (Satheeshan et al., 2020). Furthermore, Varghese et al. (2024) highlighted the use of VCO in cooking and hair care in Sri Lanka. Research on the health benefits of VCO has grown rapidly in the past 10 years particularly in China. Consequently, more than 30 brands of VCO exist in Hainan Province alone. The growing demand for VCO is attributed to its abundance of bioactive compounds, including MCFAs and polyphenols, which may exhibit anti-inflammatory, antioxidant, and antimicrobial properties (Fig. 1). However, it is important to note that most of these reported effects remain proposed, as human studies remain limited and findings are not always consistent. These compounds may provide various benefits, such as supporting cardiovascular health, improving skin barrier function, and helping in weight management (Chew, 2019; Ma & Lee, 2016). Furthermore, a study has shown that VCO possesses neuroprotective properties, which may positively impact disorders such as Alzheimer's disease (AD) (Demirel et al., 2024). The increasing use of VCO in the formulation of functional foods and nutraceuticals demonstrates its growing demand as a health-promoting ingredient.

### 4.1 Antioxidant activity

The high contents of MCFAs and different phenolic compounds present in VCO mainly contribute to its antioxidant effects. MCFAs can be passively transported to mitochondrial metabolism where their metabolites particularly ketone bodies play a main role in preventing oxidative stress (Ramesh et al., 2021). The interactions made with dietary fats and gut microbiota modulate the antioxidant activity of virgin coconut oil phenolics (VCOP). In addition, the metabolites in the form of antioxidant components directly enter the bloodstream and circulate to various organs and tissues.

**Fig. 2:**
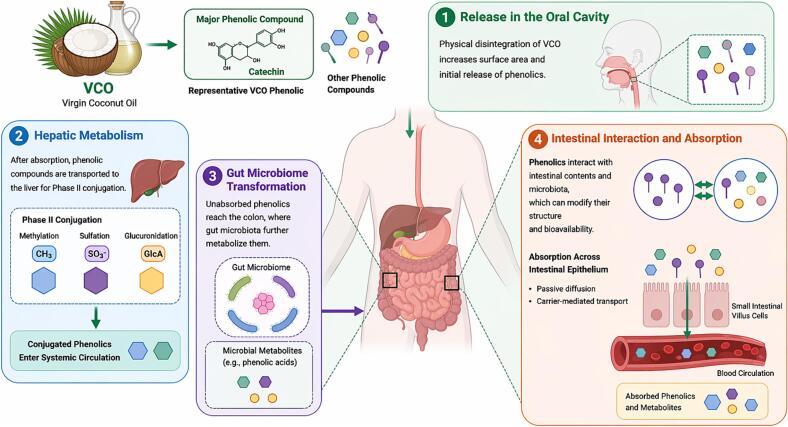
Schematic illustration of the metabolism and absorption of VCO phenolic compounds in the human body. Following ingestion, phenolic compounds (e.g., catechin and related polyphenols) are released during digestion and interact with the gastrointestinal environment. These compounds undergo biotransformation by the gut microbiota, generating smaller phenolic metabolites with enhanced bioavailability. Absorbed phenolics and metabolites are transported across the intestinal epithelium into systemic circulation and subsequently subjected to hepatic phase II metabolism (methylation, sulfation, and glucuronidation). The resulting conjugated metabolites circulate in the bloodstream, contributing to the biological activity of VCO.

The antioxidant mechanism of VCOP is associated with the digestion and absorption processes in the body. To fully understand these mechanisms requires identifying the relevant pathways involved, as illustrated in Fig. 2. The small and large intestines mainly digest and absorb phenolic compounds. These compounds can be generally classified based on their molecular size into phenolic acids and complex phenolic compounds. Phenolic acids (e.g., rosmarinic acid (RA), ferulic acid (FA), P-CA, vanillic acid (VA), syringic acid (SA), and caffeic acid (CA)) found in VCOP are simple phenolic compounds that can be rapidly absorbed in the small intestine (Gowd et al., 2019). In addition, VCOP contains flavan-3-ols called catechins which are glycosidic flavonoids present as monomeric glycosides. These compounds enter the duodenum intact and conjugate with glucuronic acid to form glucuronic acid esters. Then, the glucuronic acid esters are transported to the large intestine, where microbial esterases break them down into distinct acids. These metabolites are transported through the hepatic portal vein to the liver where they undergo further modification through glucuronidation, methylation, and sulfation before being released into systemic circulation. Upon ingestion, VCOP enters the small and large intestines where it is broken down into small phenolic acid molecules that enable antioxidant activity in various organs. They are carried to the bloodstream through the liver and as they circulate throughout the body their antioxidative properties become evident in different tissues and organs.

**Fig. 3:**
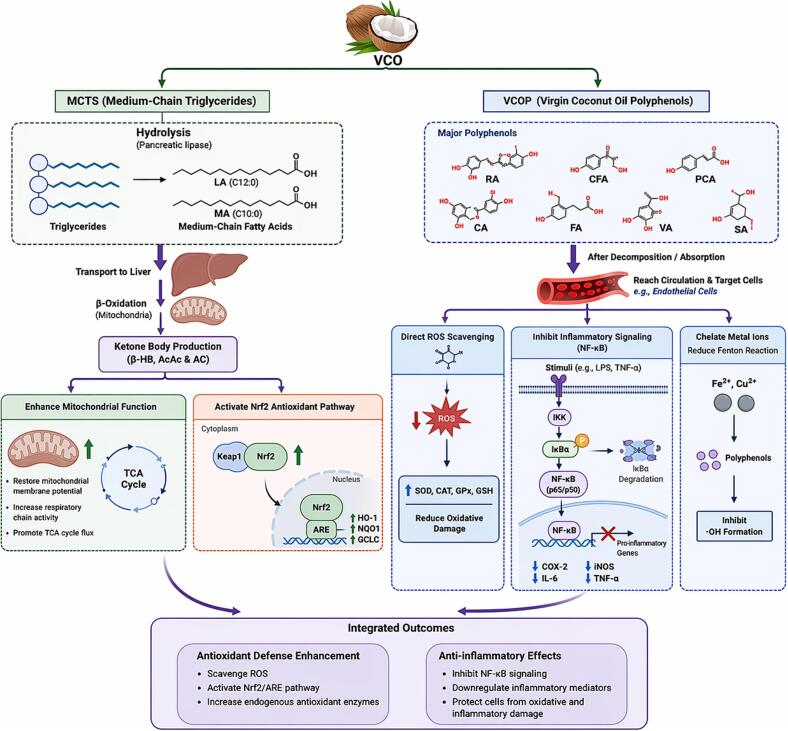
Schematic antioxidant mechanism of VCO. Medium-chain triglycerides (MCTs) release lauric acid (LA) and myristic acid (MA), which undergo β-oxidation to produce ketone bodies that enhance mitochondrial function and activate the Nrf2/ARE antioxidant pathway. VCO polyphenols, including rosmarinic acid (RA), caffeic acid (CFA), p-coumaric acid (PCA), catechin (CA), ferulic acid (FA), vanillic acid (VA), and syringic acid (SA), exert antioxidant effects by scavenging reactive oxygen species (ROS), upregulating endogenous antioxidant enzymes, inhibiting NF-κB–mediated inflammation, and chelating metal ions.

The antioxidant mechanisms of VCOP can be divided into four types. Firstly, VCOP serves as a hydrogen donor neutralizing reactive oxygen species (ROS) and acting as a ROS scavenger which directly inhibits their formation and prevents further oxidative damage to other compounds. Secondly, VCOP improves the endogenous antioxidant defense system which enhances the antioxidant capacity of organisms. This system includes enzymes such as catalase (CAT), superoxide dismutase (SOD), dehydroascorbate reductase (DHAR), glutathione-S-transferase (GST), and glutathione peroxidase (GPx), along with non-enzymatic components like glutathione (GSH), tocopherols, phenolic compounds, and ascorbic acid. All these components work together to inhibit the production of ROS and reduce oxidative stress (Bhuyan et al., 2020). For example, SOD catalyzes the conversion of superoxide radicals into hydrogen peroxide and oxygen (Okabe et al., 1996; Veskoukis et al., 2012), CAT decomposes hydrogen peroxide and prevents hydroxyl radical formation (Bianchi et al., 2019), GPx inhibits peroxide formation (Valko et al., 2007), while GSH, synthesized in the liver plays fundamental roles in free radical scavenging (Ramya et al., 2022). Thirdly, VCOP stimulates the endogenous antioxidant defense systems by regulating associated cellular signaling pathways, thus making a strong antioxidant effect. According to studies, different phenolic compounds in VCO have been shown to modulate key inflammatory mediators and signaling components, including the downregulation of COX-2 (an inflammatory enzyme), PGE2 (a pro-inflammatory eicosanoid), and transcriptional regulators such as AP-1 and MMP-1, and NADPH oxidase 4 (Nox4) (Ha et al., 2018; John & Arockiasamy, 2021). Moreover, they also enhance signaling components involved in oxidative stress defense, including the AMPK enzyme, antioxidant enzyme HO-1, nuclear receptor PPARγ, chaperone protein Hsp27, and the Nrf2 transcription factor pathway, resulting in an improved antioxidative response (Hitl et al., 2021; Jing et al., 2018; Tsai et al., 2019). Lastly, VCOP chelates/reduces metal ions to inhibit the production of toxic free radicals. This characteristic is mainly due to the polyhydroxy structure of VCOP (Alu'datt et al., 2018; Olszowy, 2019). The antioxidant mechanism of VCO is illustrated in Fig. 3, while the antioxidant activity of VCO is shown in Table 1.

**Table 1:** Antioxidant activity of VCO

References	Study Type	Sample Type	Extraction Method	Antioxidant Assay	Findings
(Syafitri et al., 2023)	*In vitro*	Commercial VCO samples	Tested commercial VCO	FRAP, ABTS, CUPRAC, DPPH	Demonstrated significant antioxidant activity across multiple assay methods; VCO showed consistent radical scavenging capacity
(Lakshmi et al., 2024)	*In vitro*	VCO-mediated ZnO nanoparticles	VCO-assisted green synthesis (using VCO as reducing and capping agent)	DPPH, ABTS, FRAP	VCO-synthesized ZnO nanoparticles exhibited strong antioxidant activity, with IC₅₀ values of 78.99 μg/mL (DPPH), 51.46 μg/mL (ABTS), and 4.68 μg/mL (FRAP), indicating high radical-scavenging and reducing potential
(Librado & Von Luigi, 2013)	*In vitro*	Phenolic extract from VCO	Phenolic fractionation, HPLC profiling, Folin–Ciocalteu (TPC determination)	DPPH, TBARS, nitric oxide (NO•) scavenging, hydroxyl radical (•OH), H₂O₂ modulation, alamarBlue® cell viability	VCO phenolic extract showed concentration-dependent antioxidant effects, including strong DPPH radical scavenging (IC₅₀ = 14.9 mg/mL), inhibition of lipid peroxidation (TBARS IC₅₀ = 0.432 mg/mL GAE), and suppression of nitric oxide radicals (IC₅₀ = 0.023 mg/mL GAE). It also modulated hydroxyl radical and H₂O₂ formation and reduced Hep-G2 cell viability
(Illam et al., 2022)	*In vitro* and *in vivo*	Whole VCO, polyphenolic fraction (VCOP), non-phenolic oil fraction (VCOF)	Fresh kernel extraction (no chemical refining), fractionation	Cell ROS (DCF fluorescence), cell survival assays, hepatic antioxidant markers (catalase, SOD), lipid peroxidation	VCOP fraction showed superior antioxidant activity; significantly reduced fluoride-induced oxidative stress and improved hepatic antioxidant enzyme activities
(Illam et al., 2017)	*In vitro*	Polyphenolic fraction (VCOP) from VCO	LC/MS profiling of VCOP	Cell-based oxidative stress assays (H₂O₂, AAPH challenges), GSH, GR, GPx, GST, lipid peroxidation	VCOP provided dose-dependent cellular protection against oxidative stress; enhanced glutathione-related antioxidant enzyme activities
(Karunasiri et al., 2020)	*In vitro* and *in vivo*	Phenolic extract from coconut oil meal	Phenolic extraction from coconut oil meal by-product	DPPH, ABTS, FRAP, CUPRAC*, in vivo* antioxidant enzyme activities	Coconut oil meal phenolics demonstrated strong antioxidant capacity and protected against oxidative damage in animal models
(Leliqia et al., 2020)	*In vitro*	Methanol fraction of VCO combined with honey	Methanol extraction from VCO	DPPH	Synergistic antioxidant effects observed when VCO was combined with honey; enhanced radical scavenging activity
(Mansor et al., 2012)	*In vitro*	VCO samples from different processing methods	Cold centrifugation, hot extraction, enzymatic methods	Phenolic content analysis, comparative antioxidant evaluation	Cold centrifugation preserved higher phenolic content and antioxidant activity compared to heat-based extraction methods
(Pengseng et al., 2013)	*In vitro*	VCO in model lipid systems	VCO application in lipid systems	DPPH, ABTS, peroxide value (PV), TBARS, p-anisidine under thermal processing	VCO maintained antioxidant activity under thermal processing conditions; effectively prevented lipid oxidation in food systems
(Gani et al., 2020)	*In vitro*	β-glucan stabilized VCO nanoemulsion ± EGCG or α-tocopherol	VCO nanoemulsion preparation with β-glucan stabilization	DPPH, FRAP after simulated gastrointestinal digestion	Nanoemulsion formulation enhanced antioxidant stability during digestion; combination with EGCG or α-tocopherol showed additive effects
(Penprapai & Intharit, 2017)	*In vitro*	Coconut oil enriched with curcuma extracts	Enrichment with curcuma species extracts	Total phenolic content, antioxidant activity assays	Curcuma enrichment significantly increased total phenolic content and antioxidant capacity of coconut oil

The antioxidant potential of VCOP is attributed to their ability to act through multiple mechanisms, including direct scavenging of ROS, enhancement of endogenous antioxidant enzyme systems, modulation of redox-sensitive cellular signaling pathways, and metal ion chelation. Collectively, these actions support systemic antioxidative activity and contribute to the maintenance of redox homeostasis. Accordingly, the incorporation of VCO in the diet may support cellular antioxidant defenses and promote redox balance, thereby contributing to overall physiological resilience.

### 4.2 Dermatological activity of VCO

#### 4.2.1 Anti-inflammatory and skin barrier effects

VCO possesses strong anti-inflammatory properties and is beneficial for both dietary supplementation and topical application. These effects are primarily attributed to their high content of MCFAs (e.g., lauric, capric, and caprylic acids) and phenolic compounds, which act to reduce inflammation at the cellular level. In animal models, oral VCO supplementation at 500-1000 mg/kg body weight for 12 days significantly suppressed pro-inflammatory cytokines including TNF-α, IL-1β, and IL-6, improving DSS-induced inflammatory bowel disease and reducing systemic inflammation in LPS-challenged BALB/c and Swiss albino mice (Prabha et al., 2024). Complementary *in vitro* studies using LPS-stimulated RAW 264.7 murine macrophages demonstrated that VCO inhibited gene expression of iNOS, COX-2, TNF-α, IL-1β, and IL-6, with significant differences compared to controls (p < 0.05) (Amin et al., 2020). Similarly, hot-pressed VCO (HVCO) at 31.5 and 62.5 μg/mL maintained high cell viability (>90%) and downregulated inflammatory genes, attributed to its higher phenolic content (Nasution et al., 2023).

The anti-inflammatory effects of VCO have also been demonstrated in nephrotoxicity and inflammation models. In rats, dietary VCO at 5-15% prior to methotrexate (MTX) administration mitigated oxidative stress, restored renal antioxidant enzymes, increased GSH, and reduced IL-6, nitric oxide, and CRP, with histopathological improvements (Famurewa, Akunna, et al., 2020). Similarly, 16-day VCO supplementation in gentamicin-induced nephrotoxicity improved renal function, enhanced antioxidant activity, increased GSH, and downregulated pro-inflammatory markers including NO, iNOS, NF-κB, and caspase-3 (Famurewa et al., 2020). Moreover, HVCO with higher phenolic content (40.03 ± 5.8 μg/mL) showed superior nitric oxide scavenging activity (IC₅₀ = 14.84 ± 0.81 μg/mL) compared to fermentation-processed VCO (IC₅₀ = 29.41 ± 1.7 μg/mL). In DSS- and formalin-induced acute and chronic inflammation models, HVCO closely approached the standard anti-inflammatory effect of diclofenac (Illam et al., 2021).

**Fig. 4:**
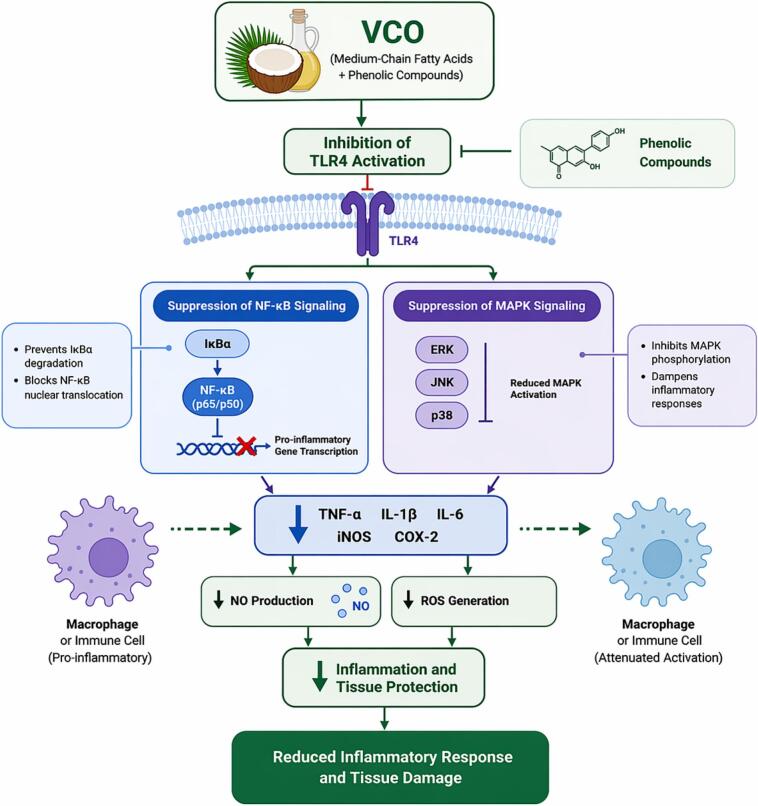
Schematic representation of the anti-inflammatory mechanisms of VCO. VCO components (medium-chain fatty acids and phenolic compounds) inhibit TLR4 activation, leading to suppression of downstream NF-κB and MAPK signaling pathways. This results in decreased expression of pro-inflammatory mediators (TNF-α, IL-1β, IL-6, iNOS, COX-2), reduced nitric oxide (NO) and reactive oxygen species (ROS) production, and modulation of macrophage activation. These coordinated upstream and downstream regulatory events contribute to attenuation of inflammation and protection against tissue damage.

Additionally, VCO showed significant anti-inflammatory effects in obesity and neuroinflammation models. In an *in vivo* study, male BALB/c mice fed a high-carbohydrate (HC) diet for eight weeks followed by VCO supplementation at 1000, 3000, or 9000 mg/kg body weight for 4 weeks exhibited reduced adiposity, improved glucose tolerance, lowered serum glucose and lipid levels, and decreased adipose tissue TNF-α and IL-6 concentrations, suggesting amelioration of obesity-induced inflammation (Zicker et al., 2019). Furthermore, in both *in vitro* and *in vivo* models, VCO demonstrated neuroprotective effects against LPS-induced neuroinflammation. *In vitro*, VCO (100 μg/mL) enhanced SK-N-SH cell viability by 57% and reduced ROS by 31%, while *in vivo* supplementation in Wistar rats (1-10 g/kg/day for 31 days) improved spatial memory and decreased markers of oxidative stress and inflammation in the brain, indicating its potential against neurodegenerative conditions (Rahim et al., 2021). Importantly, human clinical evidence also supports VCO’s systemic anti-inflammatory effects. In a randomized crossover clinical trial involving twelve postmenopausal women, VCO intake for 28 days significantly increased HDL (+6.6 ± 7.5 mg/dL) together with a modest rise in LDL (+13.5 ± 16.0 mg/dL), while reducing IL-1β levels among individuals with detectable samples, indicating a mild anti-inflammatory response. These findings provide preliminary quantitative evidence suggesting that VCO exert systemic anti-inflammatory effects in humans (Harris et al., 2017). The anti-inflammatory pathway of VCO, illustrating its effects on TLR4 inhibition, NF-κB/MAPK suppression, cytokine downregulation, and antioxidant activation, is presented in Fig. 4.

In addition to its systemic anti-inflammatory effects, VCO has demonstrated notable benefits for skin barrier function. In a clinical study, topical application of VCO and structured VCO (SVCO) creams (30% w/w oil and 5% w/w α-tocopherol) significantly enhanced skin hydration, reduced transepidermal water loss (TEWL), and improved elasticity compared to baseline (Ibrahim et al., 2022). Complementary *in vitro* experiments using human THP-1 monocytes and HaCaT keratinocytes showed that VCO at non-cytotoxic doses (∼700-800 μg/mL) suppressed pro-inflammatory cytokines TNF-α, IFN-γ, IL-6, and IL-8, while upregulating barrier proteins such as involucrin and filaggrin, indicating enhanced skin protection and hydration (Varma et al., 2019).

Moreover, various clinical studies have demonstrated VCO’s therapeutic potential in skin-related disorders. In a descriptive human case study involving a type II diabetic patient with pruritus, application of VCO twice-daily during massage for 3 days reduced skin dryness and itching severity as evaluated by Overall Dry Skin Score (ODSS) and Itch Numeric Rating Scale (NRS) (Zamroni et al., 2023). Similarly, in a randomized, double-blind, pilot study of pediatric patients with atopic dermatitis (n=29), a 20% VCO cream applied twice daily for 4 weeks significantly reduced Severity Scoring of Atopic Dermatitis (SCORAD) scores compared to a commercial emollient by improving eczema symptoms and skin hydration (Castro & Apostol, 2020). Furthermore, a randomized, double-blind clinical trial in 117 pediatric patients with mild to moderate atopic dermatitis demonstrated that topical VCO significantly reduced disease severity, with a 68.23% reduction in SCORAD index compared to 38.13% with mineral oil (*P* < 0.001). Quantitative assessments further confirmed the superior efficiency of VCO over mineral oil in improving epidermal barrier function, strengthening the clinical evidence supporting VCO’s role in enhancing skin barrier integrity (Evangelista et al., 2014). Furthermore, in diabetic rats (n=30, divided into six groups), topical application of VCO combined with black cumin oil for 14 days enhanced wound healing, as evidenced by faster wound closure, increased collagen deposition, and reduced TNF-α expression (Arman et al., 2024).

In recent years, innovative applications of VCO for skin protection have been explored through various experimental models. In a laboratory-based *in vitro* study, a smart, photo-responsive multilayer drug delivery system was developed using a layer-by-layer (LbL) method by incorporating chitosan, polyoxometalate salt, VCO, and curcumin. Hollow capsules (2-5 μm) were dispersed into chitosan/VCO emulsions, molded, and dried at 37 °C for 48 hours. The system achieved a gradual release of bioactives (approximately 37% at 48 hours) and enhanced antioxidant activity (maximum 38.1%), although no animal or human models were involved, highlighting potential for future topical skin barrier therapeutics (Rodrigues et al., 2024).

Moreover, in human clinical settings, a large randomized controlled trial conducted on preterm infants (n=2294; Group A: 1146 received VCO application, Group B: 1148 received massage without oil) investigated the effects of daily topical VCO use from birth until 28 days. Assessments at days 7, 14, 21, and 28 showed that VCO significantly improved skin maturity, reduced incidences of hypothermia and apnea, and enhanced neurodevelopmental outcomes evaluated at 3, 6, and 12 months, without notable adverse effects, supporting its safety and effectiveness as an emollient (Konar et al., 2020). Furthermore, a quasi-experimental study involving human uremic patients undergoing hemodialysis (n=80, divided into two groups) compared the impact of VCO application versus chemical-free lotion. Over a treatment period, VCO significantly improved skin moisture (p<0.05), although the chemical-free lotion, applied twice daily, demonstrated slightly better normalization of skin hydration without excessive oiliness (Saodah et al., 2020). Collectively, these findings strengthen the multifunctional properties of VCO, including its anti-inflammatory, antimicrobial, and skin-barrier protective effects, relevant for managing conditions such as eczema, dermatitis, and wound healing (Chew, 2019). The detailed effects of VCO on skin barrier function and its anti-inflammatory properties are presented in Table 2. Several clinical studies have demonstrated the efficiency and safety of VCO in improving skin barrier function across diverse populations. A randomized, double-blind trial in 34 patients with mild to moderate xerosis showed that VCO was as effective as mineral oil in improving skin hydration and lipid levels, with no significant differences in TEWL or pH, while exhibiting a slightly better trend in subjective assessments (Agero & Verallo-Rowell, 2004). Similarly, an assessor-blinded trial in elderly patients with mild to moderate senile xerosis reported that twice-daily VCO application for two weeks significantly improved skin dryness, hydration, and lipid content, as reflected by reductions in ODSS and increases in corneometer and sebumeter readings, without adverse effects (Alas et al., 2014). Moreover, in pregnant women, topical VCO significantly reduced the incidence of striae gravidarum (25% vs. 45%, p < 0.05) and lowered SGAS scores at 32 weeks (1.8 ± 0.4 vs. 2.6 ± 0.5, p < 0.05) and postpartum (1.4 ± 0.3 vs. 2.2 ± 0.4, p < 0.01), supporting its effectiveness and safety for stretch mark prevention (Ulya et al., 2024). In elderly participants, plant oil mixtures reduced clinical dry scores and pruritus severity while increasing hydration and sebum levels at weeks 2 and 4 (p < 0.001) compared to VCO, although both treatments were safe and well-tolerated (Yahya et al., 2021). Additionally, in hemodialysis patients, VCO significantly improved skin moisture compared to controls (P < 0.001), highlighting its potential as a safe alternative for managing dry skin in chronic kidney disease (Pitriani et al., 2020).

**Table 2:** Skin barrier and anti-inflammatory activity of VCO.

References	Study Type	Sample Type	Skin Barrier Activity	Anti-Inflammatory Activity	Key Findings
(Varma et al., 2019)	*In vitro*	VCO applied to cultured skin cells	Enhanced markers of skin protection and barrier-related endpoints in cell models	Suppression of inflammatory markers *in vitro*; reduced pro-inflammatory responses in treated cells	VCO demonstrated protective effects on skin cells with reduced inflammatory signaling, supporting topical dermatological applications
(Pham et al., 2022)	Animal (mouse model)	Topical anti-aging serum with coconut oil base + deer antler stem cell extract (0.01-2.5%)	Increased epidermal thickness and enhanced collagen density at treated sites compared to controls	Indirect anti-inflammatory/anti-aging effects via improved hydration and structural recovery	Coconut oil-based formulations significantly increased collagen density and epidermal thickness, supporting use in skin repair and anti-aging applications
(Kim et al., 2017)	Human skin model/*ex vivo*	Cultured coconut extract (CCE) applied topically to human skin samples	Upregulation of skin barrier molecules and collagens; improved barrier-related markers on human skin samples	Decreased inflammatory readouts and modulation of barrier protein expression; increased structural protein expression	CCE enhanced barrier molecule and collagen expression in human skin models while demonstrating anti-inflammatory effects
(Vaughn et al., 2018)	Systematic review	Topical VCO and other plant oils	Improved transepidermal water loss (TEWL) and skin capacitance measurements in reviewed studies	Decreased TEWL and modulated inflammation through fatty acid effects on barrier lipids and antioxidant actions	Synthesized evidence showing VCO can enhance barrier repair and reduce inflammation when applied topically
(Zidni et al., 2022)	Systematic review and meta-analysis	VCO application in preterm infants	Improved skin integrity scores and barrier function in neonatal populations	Reduced skin inflammation and irritation in vulnerable neonatal skin	Meta-analysis demonstrated effectiveness of VCO in improving skin integrity in preterm infants with significant clinical benefits
(Evangelista et al., 2014)	Human clinical trial	VCO vs. mineral oil in atopic dermatitis (34 patients)	Significant improvement in skin barrier function and SCORAD index	Reduced inflammation and colonization of *Staphylococcus aureus*	VCO superior to mineral oil in treating mild to moderate atopic dermatitis with anti-inflammatory benefits
(Verallo-Rowell et al., 2008)	Human clinical trial	VCO vs. virgin olive oil in atopic dermatitis (26 patients, pediatric)	Improved skin barrier function and reduced dryness	Significant anti-inflammatory effects and reduced disease severity	VCO more effective than olive oil in pediatric atopic dermatitis with superior anti-inflammatory properties
(Intahphuak et al., 2010)	Animal (rats)	Topical VCO on wound healing	Accelerated wound closure and enhanced collagen synthesis	Reduced inflammatory cell infiltration and faster resolution of inflammation	VCO significantly enhanced wound healing with improved barrier restoration and reduced inflammation
(Nevin & Rajamohan, 2010)	*In vitro* antimicrobial	VCO against skin pathogens	Indirect barrier protection through antimicrobial activity	Anti-inflammatory effects via pathogen elimination	VCO demonstrated broad-spectrum antimicrobial activity against common skin pathogens

Complementing these findings, VCO has shown anti-inflammatory and barrier-protective effects in clinical and preclinical studies. In a randomized, double-blind study of 91 participants, VCO reduced mosquito bite inflammation, with mean lesion sizes of 0.02 cm in the VCO group versus 0.71 cm in the hydrocortisone group by day 3, and 34.09% of VCO participants achieved total lesion clearance within the first hour, without adverse effects (Ulya et al., 2018). In a preclinical study of 28 pregnant mice, a VCO nanoemulgel (150 μg/mL) significantly reduced TEWL (mean = 8.90 vs. 23.90, p < 0.05) and striae gravidarum scores (mean = 5.00 vs. 15.14, p < 0.05), with a positive correlation between TEWL and striae severity (r = 0.483, p < 0.05), highlighting its potential to prevent barrier dysfunction (Pratami et al., 2025). Collectively, these studies provide robust quantitative evidence supporting VCO may be a safe and effective moisturizer with skin barrier-enhancing, anti-inflammatory, and protective properties across clinical and experimental models.

VCO has demonstrated pronounced anti-inflammatory and skin barrier–supporting effects through both dietary and topical applications. Preclinical studies consistently report reductions in pro-inflammatory cytokines, attenuation of oxidative stress, and improved tissue integrity across diverse experimental models, including obesity, neuroinflammation, and nephrotoxicity. Clinical investigations further report meaningful improvements in skin hydration, elasticity, barrier function, and symptom severity in conditions such as atopic dermatitis, pruritus, xerosis, and impaired wound healing. Collectively, these findings highlight the broad therapeutic relevance of VCO and support its role as a functional dietary component and topical agent with anti-inflammatory and barrier-enhancing properties across a range of physiological and dermatological contexts.

#### 4.2.2 Wound healing effects

The wound healing process involves five essential stages: inflammation, neovascularization, granulation tissue formation, re-epithelialization, and remodeling of the extracellular matrix (Nevin & Rajamohan, 2010; Zulkefli et al., 2023). Plant-based products such as VCO due to their antioxidant, anti-inflammatory, and antimicrobial properties, have gained attention for their wound healing potential. VCO, rich in LA and flavonoids, exhibits antibacterial, antioxidant, analgesic, and anti-inflammatory properties that support tissue repair. Several animal-based studies have demonstrated the effectiveness of ozonated and hydrolyzed VCO in enhancing wound healing parameters. In a controlled *in vivo* study involving 50 diabetic male Wistar mice (with five treatment groups) and 10 non-diabetic controls, ozonated VCO with different ozone exposure times (0 min, 90 min, 7 h, and 14 h) was applied topically. The 14-hour ozonated VCO group showed the most significant wound contraction and higher expression of healing biomarkers such as HSP90β, VEGF-A, EGF, bFGF, and CD34, indicating duration-dependent tissue regeneration (Yuniati et al., 2021). Similarly, in a randomized parallel-controlled study on 40 Sprague Dawley rats undergoing autologous skin grafts, topical application of ozonated VCO at 50.4, 103.2, and 204 mg/mL significantly increased fibroblast numbers and VEGF expression on days 6 and 12 in a dose-dependent manner, supporting its role in quickening critical phases of graft healing (Napitupulu et al., 2021). Furthermore, a randomized post-test-only experimental study using Wistar rats with second-degree burns demonstrated that hydrolyzed VCO at concentrations of 70% and 100%, applied for 6 to 12 days, significantly enhanced VEGF expression and collagen thickness compared to base cream, emphasizing its proficiency in vascularization and tissue remodeling during burn wound repair (Syarif et al., 2021).

In addition to experimental animal studies, clinical and quasi-clinical investigations have strengthened the wound healing potential of VCO. A quasi-experimental clinical study on 16 diabetic ulcer patients at Dr. Rasidin Hospital, Padang, Indonesia, divided subjects into two groups (n = 8), where the intervention group received 0.9% NaCl along with VCO for four days. VCO was applied topically using a stimulation technique. The results showed a significant reduction in wound surface area in the VCO-treated group compared to controls (p = 0.033), indicating that VCO may help healing in chronic ulcers through its bioactive properties (Dafriani et al., 2020). Moreover, a quasi-experimental study on male Wistar rats with contaminated excisional wounds evaluated the effectiveness of clove oil diluted in VCO. Rats received daily topical treatment for 16 days with varying concentrations of the formulation. The 5% clove oil in VCO group showed the most significant reduction in wound area (p < 0.001), highlighting the synergistic effects of VCO when combined with other plant-derived compounds with antiseptic and anti-inflammatory activities (Primandaru et al., 2023).

Overall, available evidence demonstrates that VCO supports wound healing through its influence on key biological processes, including fibroblast activity, angiogenesis, and extracellular matrix remodeling. Beneficial effects have been observed across a wide range of wound models, including diabetic wounds, burn injuries, contaminated wounds, and skin grafts, highlighting the broad relevance of VCO in tissue repair. Experimental studies consistently show enhanced wound closure, improved tissue regeneration, and favorable modulation of healing-related pathways following VCO application. Human investigations further indicate that VCO-based interventions contribute to improved wound outcomes within clinical and applied settings. Collectively, these findings establish a strong biological and translational foundation supporting the role of VCO as a functional therapeutic agent for wound management. The accumulated evidence underscores the relevance of VCO in promoting tissue repair and skin regeneration, reinforcing its potential utility in dermatological and wound-care applications.

### 4.3 Effects on cardiovascular health

CVDs encompass a range of disorders affecting the heart and blood vessels, including coronary artery disease, myocardial infarction, and hypertension, and remain a leading global cause of mortality (Robinson, 2021; Saiz et al., 2022). Major contributors to CVD development include dyslipidemia, chronic inflammation, and oxidative stress. In this context, VCO has attracted increasing scientific interest for its potential role in supporting cardiovascular health. Rich in MCFAs and antioxidant compounds, VCO has been shown to influence lipid metabolism, inflammatory status, and endothelial function, thereby contributing to cardiovascular protection (Azubuike-Osu et al., 2021; Sinaga et al., 2021).

In a human crossover trial involving patients with acute coronary syndrome (ACS), VCO supplementation resulted in significant reductions in serum lipid levels and high-sensitivity C-reactive protein (hs-CRP) (p < 0.001), with small to moderate effect sizes that were both statistically and clinically meaningful (Chan et al., 2022). These findings indicate a potential beneficial impact of VCO on lipid-associated and inflammatory cardiovascular risk markers however, these results should be interpreted with caution due to limitations such as small sample size and short study duration. The effects of VCO on various cardiovascular parameters are summarized in Table 3.

**Table 3:** Effects of VCO on cardiovascular health.

References	Study Type	Sample Type	Cardiovascular Effects of VCO	Key Findings
(Chinwong et al., 2017)	Randomized open crossover clinical trial (humans)	Healthy volunteers (n=32) consuming 25mL VCO daily for 8 weeks	Lipid profile, anthropometric measures	HDL-C increased by 5.72 mg/dL (p=0.026) compared to baseline. No significant changes in LDL-C, total cholesterol, triglycerides, or anthropometric measures.
(Harris et al., 2017)	Randomized controlled trial (humans)	Adults with metabolic syndrome (n=116) consuming 30mL coconut oil daily for 12 weeks	Lipid profile, fasting glucose, blood pressure, waist circumference	Decreased triglycerides and fasting glucose. Increased LDL-C and total cholesterol compared to control group. Mixed effects on cardiovascular risk markers.
(Teng et al., 2020)	Systematic review and meta-analysis	Multiple RCTs with coconut oil interventions	HDL-C, LDL-C, total cholesterol, triglycerides	HDL-C increased (pooled MD +3.28 mg/dL). LDL-C effects variable - increased vs plant oils but similar to olive oil in some studies. Triglyceride effects inconsistent across studies.
(Kamisah et al., 2015)	Animal *in vivo* study	Sprague-Dawley rats fed heated palm oil ± VCO (1.42 mL/kg) for 16 weeks	Systolic blood pressure, cardiac TBARS, ACE activity, cardiac histomorphometry	VCO prevented blood pressure elevation caused by heated palm oil (112.91±1.32 vs 98.08±3.61 mmHg control; p<0.05). Reduced cardiac TBARS (lipid peroxidation). Reduced ACE activity and cardiac remodeling, suggesting antioxidant-mediated cardioprotection.
(Famurewa et al., 2018)	Animal *in vivo* study	Normal rats fed 10%-15% VCO diet for 5 weeks	Serum lipids, hepatic antioxidant enzymes, lipid peroxidation, cardiovascular risk indices	Reduced TC, TG, LDL and increased HDL (p<0.05). MDA (lipid peroxidation) decreased. SOD, CAT, GPx antioxidant activities increased. Improved cardiovascular risk indices.
(Nurul-Iman et al., 2013)	Animal *in vivo* study	Rats fed repeatedly heated palm oil ± VCO	Blood pressure, plasma nitric oxide, vascular reactivity	VCO prevented Blood Pressure elevation caused by heated palm oil. Restored plasma nitric oxide levels. Improved endothelial-dependent vascular responses. Demonstrated endothelial protection by VCO.
(Hamsi et al., 2015)	Animal *in vivo* study	Sprague-Dawley rats fed fresh or repeatedly heated VCO for 24 weeks	Blood pressure, inflammatory markers (VCAM-1, ICAM-1, CRP), thromboxane/prostacyclin ratio	Fresh VCO had neutral effects. Repeatedly heated VCO (5×, 10×) raised blood pressure. Increased TXB2, decreased PGI2. Elevated inflammatory markers (VCAM-1, ICAM-1, CRP), demonstrating that heating VCO produces pro-inflammatory effects.
(Famurewa & Ejezie, 2018)	Animal *in vivo* study	Rats exposed to cadmium ± VCO polyphenols (10-50 mg/kg)	Serum lipids, cardiovascular risk ratios, hepatic antioxidant status	VCO polyphenols prevented cadmium-induced increases in TC, TG, LDL, VLDL. Restored HDL levels. Enhanced antioxidant defenses (SOD, CAT, GSH). Reduced MDA compared to cadmium alone.
(Nevin & Rajamohan, 2004)	Laboratory *in vitro* and animal feeding study	VCO (wet-extracted) in LDL oxidation assays; animal feeding study	LDL oxidation, tissue and plasma lipids	VCO polyphenol fraction inhibited LDL oxidation *in vitro* (reduced TBARS, carbonyl formation vs other oils). In animal feeding: decreased serum and tissue lipids, increased HDL.

Endothelial function, a key determinant of vascular health, has also been shown to respond favorably to VCO intake. In a randomized trial involving healthy young adults (n = 34), four weeks of daily VCO consumption (30 mL/day) significantly increased popliteal artery flow-mediated dilation, reflecting improved endothelial responsiveness without affecting exercise-induced blood flow (Robinson et al., 2019).

Preclinical studies further support the cardioprotective properties of VCO. In male rats exposed to doxorubicin-induced cardiotoxicity, oral pre-treatment with VCO (10 mL/kg body weight for 6 days) significantly reduced cardiac injury markers such as lactate dehydrogenase (LDH) and creatine kinase-MB (CK-MB). Moreover, a combination of VCO and EVOO preserved cardiac biomarker levels close to normal values and improved myocardial histoarchitecture, indicating enhanced cardioprotection through synergistic dietary lipid interactions (Utari et al., 2022).

Clinical evidence also suggests that VCO may positively modulate lipid metabolism when used alongside lifestyle interventions or pharmacotherapy. In obese women, VCO supplementation combined with aerobic exercise for eight weeks produced significantly greater reductions in triglycerides and total cholesterol compared to exercise alone, highlighting its capacity to enhance lipid-lowering responses associated with physical activity (Sinaga et al., 2021). Additionally, in an open-label pilot study involving obese but otherwise healthy adults, four weeks of VCO supplementation significantly reduced waist circumference, particularly among male participants, suggesting favorable effects on central adiposity linked to cardiovascular risk (Liau et al., 2011).

However, not all studies report purely beneficial outcomes. In a crossover observational study with healthy, non-obese male volunteers (n = 22, aged 28–50 years), consumption of approximately 35 g/day of VCO for 8 weeks led to significant increases in total cholesterol and LDL-C levels compared to baseline, although no significant changes were observed in HDL-C, triglycerides, anthropometric measures, or inflammatory markers (Jeyakumar et al., 2023). Similarly, a comprehensive meta-analysis by Eyres et al. (2016) reported that coconut oil consumption significantly increased total cholesterol and LDL-C levels compared to cis-unsaturated vegetable oils, although the magnitude of increase was lower than that observed with butter. These findings highlight the complex and sometimes contradictory effects of VCO on lipid metabolism. While certain studies suggest improvements in HDL-C and metabolic markers, the consistent elevation of LDL-C observed in controlled trials and meta-analyses raises concerns regarding its cardiovascular implications. Therefore, these findings emphasize the need for cautious use of VCO, particularly in individuals at risk of cardiovascular disease, and underscore the importance of long-term, well-controlled studies across diverse populations.

Collectively, current experimental and clinical evidence suggests that VCO may exert beneficial effects on cardiovascular health through multiple mechanisms, including modulation of lipid-associated risk markers, attenuation of inflammatory responses, enhancement of endothelial function, and protection against cardiac injury. These effects are supported by findings from preclinical models and selected human studies across diverse populations. However, these benefits should be interpreted with caution, as VCO is rich in saturated fatty acids and has been associated with elevations in LDL-C in several controlled trials and meta-analyses. This raises concerns regarding its potential adverse effects on cardiovascular risk, particularly with excessive intake or in individuals with pre-existing metabolic or cardiovascular conditions. Therefore, while VCO may have cardioprotective potential, its consumption should be moderated and considered within the context of overall dietary patterns and individual health status.

### 4.4 Effects on diabetes

Diabetes mellitus (DM) is a major global health concern affecting approximately 463 million individuals worldwide (Yapislar & Gurler, 2024). Chronic hyperglycemia leads to poor glycemic control and multiple complications, including delayed wound healing, which may result in severe outcomes such as amputation. VCO has attracted interest as a supportive therapy for diabetes management due to its antioxidant, anti-inflammatory, and wound-healing properties. Preclinical research has shown that VCO enhances re-epithelialization, collagen fiber deposition, and wound contraction, resulting in superior healing in diabetic rats compared to controls (Wong et al., 2019). Beyond wound care, its potential in glycemic regulation has also been explored clinically. In a quasi-experimental study, 46 pregnant women with gestational diabetes mellitus (GDM) consumed VCO (5 mL, six times daily) with a low-carbohydrate diet, leading to a significant reduction in fasting blood glucose from 155.19 mg/dL to 153.50 mg/dL (p = 0.000), suggesting its role as an adjunct non-pharmacological intervention (Saudah et al., 2021).

Complementing these findings, animal studies have provided mechanistic insights into VCO’s antidiabetic effects. In a study on 30 streptozotocin-induced diabetic rats, enzymatically hydrolyzed VCO (HVCO; 4 or 6 mL/kg BW) administered for 30 days significantly lowered blood glucose and HbA1c levels, enhanced SOD activity, and improved pancreatic insulin expression, indicating stronger antioxidant and glycemic control effects compared to regular VCO and metformin (Margata et al., 2020).

Clinically, VCO has demonstrated beneficial effects on lipid profiles in diabetic and metabolic syndrome populations. In a controlled pre-post clinical study, diabetic patients supplemented with VCO (1.2 mL/kg BW/day, divided into three doses) for 30 days showed a significant decrease in LDL levels (p = 0.002) and an increase in HDL levels (p = 0.031), along with reductions in energy and cholesterol intake (Setyawati et al., 2023). These results support that VCO’s play a role in improving cardiovascular risk factors when combined with dietary therapy. In animal models, VCO at 200 mg/kg reversed liver damage and improved lipid profiles in HFD-induced obese rats (n = 60) over four weeks, though its glucose-lowering effects were limited (Adeyemi et al., 2020).

VCO’s anti-inflammatory and antioxidant actions in diabetes have also been emphasized in several studies. In an experimental trial on 70 adult male Wistar rats, VCO (10 mL/kg BW) for two weeks significantly reduced CRP and IL-6 levels, particularly in diabetic rats exposed to atrazine, indicating its potential to attenuate systemic inflammation (Helen et al., 2024). Its antidiabetic properties are associated to LA, which stimulates pancreatic β-cell insulin production and reduces oxidative stress and inflammation (Rahmawati et al., 2023). Additionally, a VCO-enriched diet improved renal function in diabetic rats, supporting its nephroprotective role in diabetic nephropathy (Akinnuga et al., 2019).

In diabetic wound management, VCO formulations have shown promising outcomes. A study involving 30 streptozotocin-induced diabetic rats treated topically with VCO and black cumin oil showed significantly increased VEGF gene expression, enhanced wound closure, and improved tissue regeneration compared to controls (Arman et al., 2024). Similarly, topical ozonated VCO applied to 50 diabetic Wistar mice demonstrated dose-dependent wound healing effects, with 14 hours of ozonation resulting in the greatest wound size reduction and enhanced tissue repair (Yuniati et al., 2020). These findings support the potential of VCO-based topical formulations as adjunctive therapies for diabetic wounds.

Accumulating evidence from preclinical and clinical studies suggests that VCO may influence metabolic regulation, lipid homeostasis, inflammatory responses, and wound healing in diabetic conditions. Preclinical investigations generally report improvements in antioxidant status, pancreatic β-cell function, lipid parameters, and tissue repair, with proposed mechanisms including lauric acid–mediated modulation of insulin signaling, attenuation of inflammatory mediators, and upregulation of vascular endothelial growth factor (VEGF). In animal models, dietary VCO has been associated with enhanced insulin sensitivity and reduced triglyceride levels, supporting its potential metabolic benefits. However, evidence from human studies remains limited and somewhat inconsistent. While some clinical studies report improvements in fasting glucose regulation and lipid profiles, including increases in HDL-C, findings regarding LDL-C and overall cardiovascular risk are variable. Additionally, most clinical trials are short-term and involve relatively small sample sizes, limiting the generalizability of these outcomes. Therefore, although current findings indicate that VCO may contribute to improved oxidative balance, lipid metabolism, glycemic control, and wound-related outcomes, its long-term efficacy and safety in diabetic populations require further validation through well-designed, large-scale clinical studies. The diverse metabolic effects of VCO in diabetic conditions are summarized in Table 4.

**Table 4:** Effects of VCO on diabetes.

References	Study Type	Sample Type	Effects of VCO on Diabetes	Key Findings
(Setyawati et al., 2023)	*In vivo* (human)	Adults with diabetes (clinical sample)	Polyphenol-mediated antioxidant effects and modulation of lipid metabolism; VCO as dietary fat replacement affecting lipid handling	LDL decreased (p = 0.002) and HDL increased (p = 0.031) after 30 days of VCO at 1.2 mL/kg body weight/day; energy and cholesterol intake declined
(Dhanasekara et al., 2022)	Review (meta-analysis)	Interventional trials of dietary coconut fats	Coconut fats blunt postprandial insulin response leading to higher postprandial glycemia and possible long-term insulin resistance	Acute: increased glucose AUC (p = 0.046) and decreased insulin AUC (p = 0.037); Long-term: increased HOMA-IR (p = 0.049)
(Malaeb & Spoke, 2020)	Review (case report)	Single T2D patient on insulin; literature survey	Phenolic anti-inflammatory effects and potential modulation of insulin requirement in susceptible patients	66-year-old man with T2D developed recurrent hypoglycemia after initiating coconut oil supplements, requiring rapid insulin dose reduction
(Narayanankutty et al., 2016)	*In vivo* (animal)	Rats fed high-fructose diet (VCO vs copra oil)	Improved hepatic redox status, ↑GSH and antioxidant enzymes, reduced lipid peroxidation and hepatic steatosis	VCO-fed rats had 17% increase in blood glucose vs 46% in copra oil group; increased hepatic GSH and reduced lipid peroxidation
(Iranloye et al., 2013)	*In vivo* (animal)	Alloxan-induced diabetic Sprague-Dawley rats (VCO 7.5-10 mL/kg)	Antioxidant enzyme upregulation, enhanced insulin secretion, pancreatic protection	Fasting glucose: DT7.5: 132.4±6.91, DT10: 131.6±12.2 vs untreated 320.4±22.99; increased GSH, CAT, SOD; decreased MDA
(Akinnuga et al., 2014)	*In vivo* (animal)	Alloxan-induced diabetic rats (10% VCO diet)	Dietary VCO modulates lipid metabolism via polyphenols and MCFAs	3-week 10% VCO diet reduced TG, TC, LDL, VLDL (p < 0.001) and improved lipid profile in diabetic rats
(Fadlalla & Seddik, 2023)	*In vivo* (animal)	STZ-diabetic rats with gentamicin nephrotoxicity (VCO 10 mL/kg/day)	Antioxidant and anti-inflammatory effects protecting renal tissue; decreased proinflammatory cytokines	VCO treatment improved renal markers: serum urea 17.35 (ND) and 38.9 (D), creatinine 1.69 (ND) and 2.96 (D)

### 4.5 Effects on obesity

Obesity, classified by the World Health Organization (WHO) as a significant global health issue is a metabolic disorder characterized by excessive body fat accumulation. It is strongly associated with the development of serious health conditions such as diabetes, CVD, and various cancers (Alfaris et al., 2023). This condition is influenced by genetics, lifestyle, and environmental factors. VCO rich in MCFAs, have potential for weight management. Several experimental animal studies have explored the anti-obesity effects of VCO under various dietary conditions. In one study, male Wistar rats were supplemented with hot process-derived VCO for 7 weeks in an HFD-induced obesity model. This intervention significantly reduced body weight, liver weight, and epididymal fat, while improving lipid profiles by lowering triglycerides, total cholesterol, LDL-C, VLDL-C, and non-HDL-C, and increasing HDL-C. Additionally, it improved liver function markers and cardiac risk indices which supports its role as a dose-dependent strategy for obesity management (Shetty et al., 2022). Similarly, a 20-week study using 60 rats (n=10 per group) evaluated three oral doses of VCO (200, 400, and 600 mg/kg) during the final 4 weeks following HFD induction and transition to a normal diet. Notably, the 200 mg/kg dose yielded the most obvious improvements in lipid profiles and oxidative stress markers, including reduced LDL-C, triglycerides, and malondialdehyde (MDA), together with increased SOD and GPx activity. Liver histopathology and inflammatory markers (IL-6, CRP) also improved, indicating that low-dose VCO can support metabolic health and hepatic function (Adeyemi et al., 2020). Furthermore, a study by Da Silva et al. (2023) using 33 male Swiss mice fed either a standard or HFD with or without low-dose VCO supplementation showed improved LDL cholesterol levels in the HFD+VCO group, without significant changes in obesity indicators or hepatic and renal health, suggesting lipid-lowering effects at low doses, but limited impact on overall obesity.

Several experimental studies highlight the anti-obesity and metabolic regulatory potential of VCO. In a study conducted by de Vasconcelos et al. (2022), male Wistar rats fed a cafeteria diet for 16 weeks and subsequently supplemented with 3000 mg/kg/day of extra virgin coconut oil (E-VCO) exhibited significant reductions in body mass, adiposity index, and hepatic fat accumulation, alongside improved insulin and leptin regulation. These findings indicate a favorable modulation of energy balance and lipid metabolism. Similarly, Zicker et al. (2019) demonstrated that BALB/c mice exposed to a high-refined carbohydrate diet and subsequently treated with VCO at doses of 1000, 3000, or 9000 mg/kg for four weeks showed consistent reductions in adiposity, improved glucose tolerance, and decreased pro-inflammatory cytokines (TNF-α and IL-6), together with attenuation of hepatic steatosis. Notably, these beneficial metabolic effects were observed across all tested doses, supporting a dose-independent protective role of VCO in obesity-related metabolic dysfunction.

Collectively, these findings demonstrate that VCO and enzymatically modified VCO (E-VCO) positively influence obesity-related parameters, including body weight regulation, lipid metabolism, insulin sensitivity, and inflammatory status. Bioactive constituents of VCO, particularly MCFAs and polyphenolic compounds such as lauric acid, contribute to its antioxidant, anti-inflammatory, and lipid-modulating actions, thereby supporting the attenuation of chronic low-grade inflammation associated with obesity (Adeyemi et al., 2020; de Vasconcelos et al., 2022). Preclinical studies consistently report reductions in adiposity, improvements in lipid handling, enhanced antioxidant enzyme activity, and downregulation of pro-inflammatory markers following VCO or E-VCO intake. These beneficial effects are especially evident when VCO is incorporated into dietary patterns that promote metabolic balance.

Human studies further support the role of VCO in obesity-related metabolic regulation, with reported improvements in anthropometric measures such as waist circumference and favorable modulation of metabolic indicators. Together, these findings highlight VCO as a functional dietary fat with relevance for metabolic health. A summary of the anti-obesity effects of VCO is presented in Table 5.

**Table 5:** Anti-obesity effects of VCO.

References	Study Type	Sample Type	Anti-Obesity Effects	Key Findings
(Liau et al., 2011)	*In vivo* human (open-label pilot)	20 obese but healthy male volunteer’s	Visceral adiposity reduction	Waist circumference decreased by 2.86 cm in males only after 4-week VCO supplementation; no changes in lipid profile
(Assunçao et al., 2009)	*In vivo* human (randomized, double-blind)	40 women with abdominal obesity	Improved lipid ratios, reduced abdominal adiposity	30 mL/day coconut oil for 12 weeks increased HDL, lowered LDL:HDL ratio, and reduced waist circumference vs. soybean oil
(Vogel et al., 2020)	*In vivo* human (randomized controlled)	29 men with obesity	Lipid profile improvement without weight loss	12 mL/day extra-virgin coconut oil for 45 days increased HDL and decreased TC/HDL ratio; no anthropometric changes
(Metin et al., 2022)	*In vivo* human (acute crossover)	20 male subjects (10 normal weight, 10 obese)	Acute appetite suppression, PYY modulation	25 g extra-virgin coconut oil reduced hunger/desire to eat in normal-weight men; trended to increase PYY
(Zicker et al., 2019)	*In vivo* animal (mouse)	Male BALB/c mice on high-refined-carbohydrate diet	Reduced adiposity, improved glucose tolerance, anti-inflammatory effects	VCO (1000-9000 mg/kg) reduced adiposity, improved glucose tolerance, decreased hepatic steatosis and adipose TNF-α/IL-6 (4 weeks)
(Gao et al., 2022)	*In vivo* animal (mouse)	C57BL/6J mice on high-fat diet	Enhanced thermogenesis via brown adipose tissue activation	5% dietary coconut oil enhanced BAT thermogenesis, increased energy expenditure, improved body and fat depot weights
(Adeyemi et al., 2020)	*In vivo* animal (rat)	Experimental obesity models	Anti-obesity effects at low doses	Low dietary doses of VCO showed beneficial anti-obesity effects in experimental models
(Arunima & Rajamohan, 2012)	*In vivo* animal (rat)	Rats compared with other oils	Improved hepatic lipid metabolism	VCO improved hepatic lipid metabolism compared to copra oil, olive oil, and sunflower oil
(Ahnan & Agustinah, 2016)	*In vivo* animal (rat)	Obese rats	Anthropometric improvements, gut microbiota changes	VCO supplementation improved anthropometric parameters and changed Bacteroidetes/Firmicutes ratio

### 4.6 Antimicrobial and immunomodulatory effects

VCO exhibits notable antibacterial, antiviral, and immunomodulatory properties, primarily attributed to its high content of LA and its derivative monolaurin. Once ingested, LA is converted to monolaurin, which can disrupt the lipid membranes of various bacteria, viruses, and fungi, leading to their inactivation (Nitbani et al., 2022; Patiung et al., 2022; Widianingrum et al., 2019). These antimicrobial effects make VCO might be a potential natural agent for immune support and topical protection against infections.

#### 4.6.1 Antimicrobial activity of VCO

**Fig. 5:**
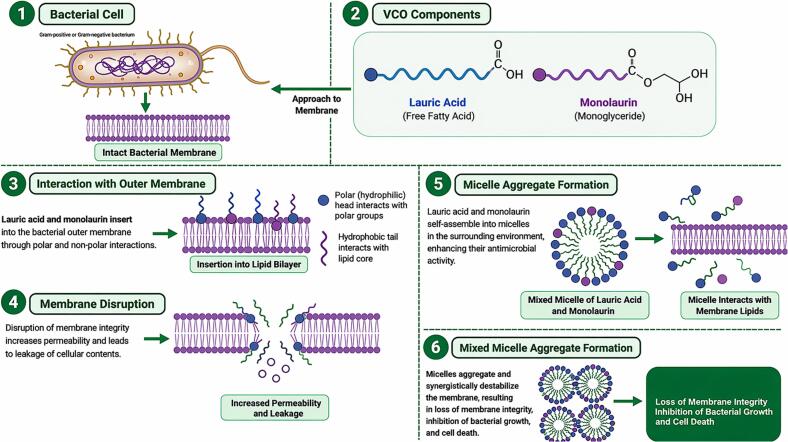
Proposed mechanism of bacterial membrane disruption by lauric acid and monolaurin derived from VCO. Lauric acid and monolaurin interact with bacterial membranes through hydrophobic and polar interactions, facilitating their insertion into the lipid bilayer. This disrupts membrane integrity, increases permeability, and leads to leakage of intracellular contents. In parallel, these compounds self-assemble into micellar aggregates that further destabilize membrane structure through cooperative interactions. These combined effects contribute to loss of membrane integrity, inhibition of bacterial growth, and eventual cell death. These mechanisms are primarily supported by in vitro and preclinical studies, and their relevance under physiological conditions requires further validation.

VCO is characterized by a high proportion of LA (approximately 48-53%), which represents its predominant fatty acid and serves as a key precursor for antimicrobial activity. While numerous studies have focused on the antimicrobial properties of isolated LA and its derivative monolaurin, it is important to emphasize that these compounds originate directly from VCO. Upon ingestion or enzymatic hydrolysis, LA present in VCO is converted into monolaurin, which contributes significantly to its biological activity. Importantly, beyond mechanistic evidence, several studies have demonstrated that VCO itself exhibits direct antimicrobial activity. For instance, VCO has been shown to inhibit the growth of *Staphylococcus aureus* by disrupting bacterial cell walls and enhancing phagocytic immune responses (Widianingrum et al., 2019). Similarly, *in vitro* studies have confirmed that VCO and coconut oil formulations exert bactericidal effects against both Gram-positive and Gram-negative bacteria, including *S. aureus* and *Escherichia coli,* through membrane damage and structural disruption (de Oliveira et al., 2018). Furthermore, antifungal activity of VCO has been reported against Candida albicans, where significant growth inhibition was observed at relevant concentrations (Tjin et al., 2016). These findings confirm that the antimicrobial effects of VCO are not only theoretical but also supported by direct experimental evidence. LA is a 12-carbon saturated fatty acid with amphiphilic characteristics arising from its hydrophobic alkyl chain and hydrophilic carboxyl group (Nitbani et al., 2016; Nitbani et al., 2022). Similarly, monolaurin has hydroxyl groups that enhance its amphiphilicity. These amphiphilic structures allow them to integrate into bacterial membranes, similar to detergent-lipid micelle interactions (Sun et al., 2022). When in polar environments, these molecules remain highly mobile and can readily penetrate lipid bilayers. Fig. 5 illustrates the membrane disruption by LA and monolaurin.

Moreover, LA and monolaurin exhibit potent antibacterial properties through membrane destabilization and increased permeability (Hassan Abd El-Ghany et al., 2024; Li et al., 2022). Their neutral nature enables diffusion through Gram-negative bacterial membranes without steric hindrance, causing leakage of cytoplasmic contents and loss of metabolic activity, ultimately leading to cell death (Kumar & Engle, 2023; Parsons et al., 2012). Against Gram-positive bacteria, their action targets the cell wall, the key structural barrier, disrupting its integrity, impairing metabolism, and allowing external compounds to penetrate more easily. This dual action enhances their broad-spectrum antibacterial potential.

**Fig. 6:**
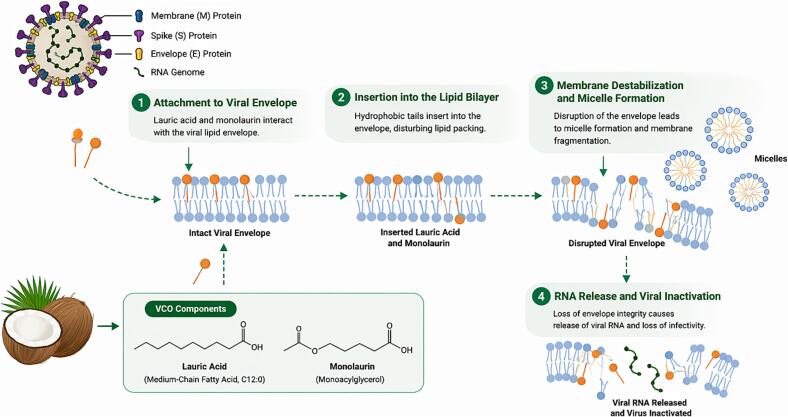
Proposed mechanism of viral membrane disruption by lauric acid and monolaurin derived from VCO. Lauric acid and monolaurin interact with the lipid envelope of enveloped viruses through hydrophobic interactions, facilitating their insertion into the viral membrane. This disrupts lipid packing, leading to membrane destabilization and micelle formation. Consequently, loss of envelope integrity results in leakage of viral RNA and inactivation of the virus.

Beyond antibacterial activity, LA and monolaurin also show antiviral properties. Both compounds can interfere with the final maturation stage of viral replication (Bartolotta et al., 2001). Their amphiphilic properties alter the fluidity and permeability of host cell membranes, weakening viral glycoprotein binding. For example, treatment with LA reduced Junin virus formation by 54.1% and increased TAG synthesis by 26.1% after 24 hours, highlighting its capacity to disrupt viral membrane host interactions. Fig. 6 summarizes the antiviral mechanism of LA and monolaurin.

The virucidal properties of LA and monolaurin arise from their lipophilic and polar characteristics (Hierholzer & Kabara, 1982; Seabra et al., 2023). Interaction with viral lipid envelopes induces vesicular aggregation and membrane disintegration, leading to lysis and cell death (Thormar et al., 1987). Additionally, LA interferes with viral protein binding to host cells, damaging the cholesterol-rich viral envelope and inhibiting replication and viral release (Hornung et al., 1994; Yasmin et al., 2023). This multifaceted mechanism supports their potential as broad-spectrum antiviral agents.

Preliminary studies suggest that MCFAs present in VCO may hold potential for prophylactic applications, such as nasal sprays or topical formulations, against respiratory viruses like SARS-CoV-2. However, despite promising *in vitro* and *in vivo* evidence, more human clinical trials are needed to establish safety, dosing, and delivery strategies for viral infections (Ramesh et al., 2021). Table 6 summarizes key antibacterial and antiviral properties of VCO and its bioactive compounds.

**Table 6:** Antibacterial effects of VCO.

References	Study Type	Sample Type	Antibacterial Effects	Key Findings
(Margata et al., 2019)	*In vitro*	Enzymatically hydrolyzed VCO (HVCO); agar disc diffusion; tested Propionibacterium acnes (ATCC 6918), *Bacillus subtilis* (ATCC 6633), *Staphylococcus epidermidis* (ATCC 12228), MRSA	50% HVCO produced inhibition zones: *P. acne* 10.08 mm, *B. subtilis* 10.53 mm, *S. epidermidis* 12.33 mm, MRSA 10.20 mm; activity was concentration-dependent; non hydrolyzed VCO inactive	Hydrolysis required: free fatty acids/monoglycerides (from HVCO) produced antibacterial activity including against MRSA
(Silalahi et al., 2014)	*In vitro*	VCO partially hydrolyzed by enzyme or NaOH; water in oil emulsions; tested *P. aeruginosa* (ATCC 25619), *S. aureus* (ATCC 29737), *S. epidermidis* (ATCC 12228*), P. acnes* (ATCC 6918)	Unhydrolyzed VCO showed no activity; hydrolyzed VCO showed antibacterial activity that increased with longer hydrolysis or higher NaOH; enzymatic hydrolysis produced stronger activity; hydrolyzed VCO was more effective against *P. aeruginosa* than other tested strains	Partial hydrolysis (generating free MCFAs and monoglycerides) is necessary to reveal antibacterial activity; enzymatic hydrolysis > alkaline hydrolysis in potency
(Loung et al., 2014)	*In vitro*	Enzymatic hydrolyzed VCO (VCOH) and hydrolyzed PKO at 25–100% (agar disc diffusion); tested *S. aureus*, *E. coli*, *S. typhi*	VCOH and PKOH showed higher antibacterial activity than non-hydrolyzed oils; activity increased with concentration; higher activity vs Gram positive (*S. aureus*) than Gram negative strains; activity was lower than standard antibiotics (chloramphenicol, tetracycline)	Hydrolysis producing lauric acid/monolaurin mixtures enhanced antibacterial effects; Gram positive bacteria were more susceptible
(Haron et al., 2018)	*In vitro*	Activated virgin coconut oil (AVCO) and laboratory VCO; MIC and MBC assays against *Streptococcus mutans*, *Lactobacillus casei*, *Candida albicans*	AVCO MIC/MBC: *L. casei* MIC 0.78 mg/ml MBC 1.56 mg/ml; *C. albicans* MIC 3.12 mg/ml MBC 6.24 mg/ml; *S. mutans* MIC 6.24 mg/ml MBC 24.96 mg/ml; VCO showed no inhibitory effect against these dental pathogens	AVCO (processed/activated) exhibited bactericidal activity with low MICs for *L. casei*; unprocessed VCO was inactive in these assays
(Niken et al., 2023)	*In vitro*	VCO (20-100% v/v) tested by Kirby Bauer disc diffusion against *Staphylococcus aureus* clinical/sample	VCO produced inhibition zones described as strong: 20% = 14.4 mm, 40% = 14.5 mm, 60% = 14.6 mm, 80% = 14.6 mm, 100% = 16.0 mm; amoxicillin control 7.9 mm; DMSO negative control 0 mm	In this assay VCO (undistilled/untreated) inhibited *S. aureus* across tested concentrations, with larger zones than the amoxicillin control used in the study
(Mahaklan et al., 2022)	*In vitro*	Co culture of *Staphylococcus epidermidis* with 2.5% (v/v) VCO; supernatant tested vs *S. aureus*; time kill assays	Supernatant from *S. epidermidis* cultured with VCO significantly reduced *S. aureus* growth compared with without VCO (p<0.05); time kill assay showed effective antimicrobial activity after 18 hours; suggests production of MCFAs during fermentation	Skin commensal lipase activity can convert VCO into antimicrobial MCFAs in situ; low VCO % (2.5%) in co culture produced inhibitory supernatant
(Purnamasari et al., 2025)	*In vitro*	VCO and palm kernel oil (PKO) tested vs *Pseudomonas aeruginosa*	VCO MIC 2048 μg/mL; MBC 4096 μg/mL (PKO MIC 4096 μg/mL MBC 8192 μg/mL); gentamicin control MIC 2 μg/mL MBC 4 μg/mL; VCO more active than PKO but far less potent than gentamicin	VCO displayed measurable activity vs *P. aeruginosa* in MIC/MBC assays but at high concentrations compared with antibiotic control

In summary, the antibacterial and antiviral activities associated with VCO are primarily attributed to LA and its derivative monolaurin, which exert their effects through membrane-disrupting mechanisms. These bioactive compounds destabilize bacterial and viral membranes, leading to cytoplasmic leakage, disruption of metabolic processes, and inhibition of viral replication. Owing to their amphiphilic nature, LA and monolaurin demonstrate broad-spectrum activity against Gram-positive and Gram-negative bacteria as well as lipid-enveloped viruses. *In vitro* and animal studies consistently demonstrate robust antimicrobial efficacy, and emerging human studies using VCO-based dietary or topical formulations report reductions in microbial colonization within practical application settings. Collectively, these findings support the role of VCO as a valuable complementary component within antimicrobial and preventive health strategies.

#### 4.6.2 Immunomodulatory effects

VCO demonstrates significant potential as an immune-enhancing agent, mainly due to its high LA content and its derivative, monolaurin. Several studies across different species, doses, and durations support its immunomodulatory effects.

In an animal study by Komatsuzaki et al. (2021), three groups of mice were fed diets containing lard, fish oil, or VCO for six weeks. VCO supplementation significantly increased IL-12 cytokine levels in the spleen and plasma IgA levels, indicating enhanced immune responses, although higher liver cholesterol was observed in VCO-fed mice. Moreover, Faradilla et al. (2020) conducted a study on chickens infected with Eimeria tenella, feeding them VCO at doses of 5, 10, and 20 mL/kg of feed for 28 days. VCO supplementation improved immune stability by maintaining normal leukocyte counts, demonstrating its role in supporting immune function in poultry. Similarly, Widianingrum and Salasia, (2021) administered VCO intragastrically (250 μL/day for one week) to female Wistar rats infected with *Staphylococcus aureus*, resulting in higher SOD activity and lymphocyte proliferation, may support immune enhancement in mammals.

Research on the broader immunomodulatory potential of VCO also includes vertebrates and invertebrates. Sagona et al. (2023) evaluated coconut oil supplementation in honeybees, finding increased fat content, improved short-term survival, and enhanced antioxidant capacity. Although phenoloxidase activity temporarily declined, these results suggest coconut oil enhances physiological resilience. Hima et al. (2020) studied young male Wistar rats fed VCO at three doses for 30 days, revealing increased splenocyte proliferation, Th1 cytokine production, improved antioxidant enzyme activity, and enhanced lipid profiles, highlighting immunomodulation via anti-inflammatory and hypolipidemic mechanisms. Furthermore, Wulansari et al. (2024) investigated the combination of Fiber Creme and VCO in doxorubicin-induced immunosuppressed rats. While no significant improvements were observed in body or spleen weight, CD4+ T cell levels and IFN-γ were significantly increased, indicating that VCO may enhance immune resilience under immunosuppressive conditions.

Collectively, current evidence demonstrates that VCO and its bioactive constituents, particularly LA and monolaurin, actively influence immune-related processes across multiple biological systems. In animal and invertebrate models, VCO supplementation has been associated with enhanced immune cell activity, strengthened antioxidant defenses, and improved resilience against infectious challenges, supporting well-defined immunoprotective mechanisms.

Human studies further indicate that VCO intake supports immune function, with reported increases in circulating IgA levels and lymphocyte proliferation, particularly when VCO is incorporated into balanced dietary patterns as part of habitual fat intake. These findings highlight the relevance of VCO within realistic dietary frameworks and underscore its role in supporting immune homeostasis. Taken together, available evidence supports the immunomodulatory potential of VCO and its bioactive components, reinforcing their functional relevance in promoting immune health across experimental and applied contexts.

### 4.7 Effects of VCO on neurological health

#### 4.7.1 Neuroprotective effects

VCO exhibits neuroprotective effects primarily due to its MCTs, which provide an alternative energy source for neurons. Mechanistically, VCO enhances acetylcholine (ACh) levels by inhibiting acetylcholinesterase (AChE), elevates antioxidant defenses (SOD, CAT, GSH), suppresses pro-inflammatory mediators (Cox-2, iNOS, NF-κB), rebalances cytokines, and reduces amyloid-β (Aβ) accumulation by inhibiting BACE-1 (Demirel et al., 2024; Irawan et al., 2022; Khalil et al., 2020).

**Fig. 7:**
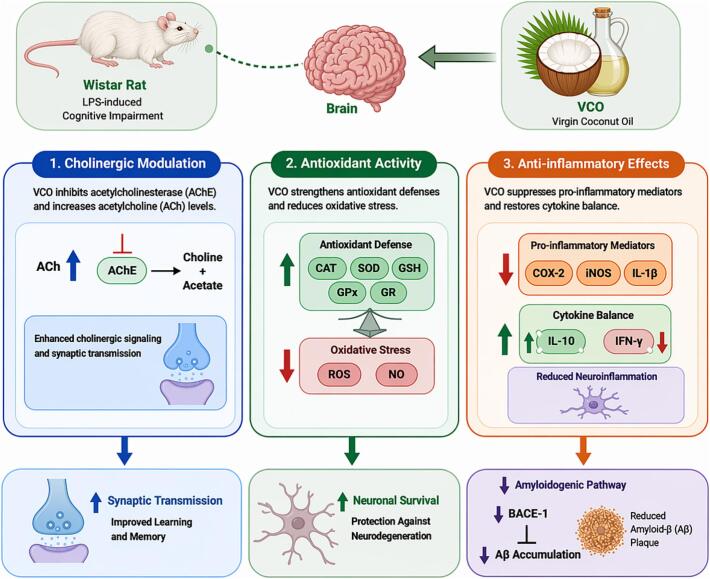
Proposed neuroprotective mechanisms of VCO in LPS-induced cognitive impairment. VCO enhances cholinergic signaling by inhibiting acetylcholinesterase (AChE), leading to increased acetylcholine (ACh) levels and improved synaptic transmission. Simultaneously, VCO strengthens antioxidant defenses (CAT, SOD, GSH, GPx, GR), thereby reducing oxidative stress markers (ROS and NO). In addition, VCO attenuates neuroinflammation by downregulating pro-inflammatory mediators (COX-2, iNOS, IL-1β) and modulating cytokine balance (e.g., increased IL-10). These coordinated effects contribute to enhanced neuronal survival and reduced amyloid-β (Aβ) accumulation, potentially via suppression of BACE-1 activity.

VCO also protects against environmental and chemical neurotoxins. For instance, rats exposed to benzene and treated with VCO showed reduced lipid peroxidation and restored antioxidant enzyme levels (Alshareef & Ibrahim, 2020). Similarly, LPS-induced neuroinflammation was mitigated by VCO, improving spatial memory, increasing ACh (+33%), decreasing AChE (-43%), reducing ROS (-31%) and MDA (-51%), and modulating cytokines (Fig. 7) (Rahim et al., 2021). VCO further ameliorated heavy metal-induced neurotoxicity: sodium arsenite exposure in rats was counteracted by VCO, restoring antioxidant enzymes, reducing MDA and NO, normalizing AChE and ADA activities, and preventing histopathological brain damage (Azubuike-Osu et al., 2021). Sodium benzoate-induced neurotoxicity was similarly mitigated, with dose-dependent improvements in cognitive performance and suppression of NF-κB (Asuku, 2022). VCO also discusses cerebrovascular benefits. In stroke-prone rats, it delayed stroke onset, reduced systolic blood pressure (∼20 mmHg), prolonged survival, and attenuated histopathological brain injury (Vitor et al., 2022). Moreover, dietary VCO upregulated tyrosine hydroxylase, NGF, SIRT1, and mTOR while inhibiting NF-κB in immune tissues, reflecting systemic neuro-immunomodulatory effects (Karrunanithi et al., 2020).

Overall, preclinical and cellular studies demonstrate that VCO engages multiple neuroprotective pathways, including modulation of cholinergic neurotransmission, enhancement of antioxidant defenses, attenuation of neuroinflammatory signaling, regulation of amyloidogenic processes, and support of cerebrovascular function. These mechanistic insights establish a strong biological foundation for the neurological benefits associated with VCO and its medium-chain fatty acid components.

Human studies further indicate that VCO consumption is associated with improvements in cognitive performance and metabolic parameters linked to brain health, particularly when incorporated into habitual dietary patterns. As a commonly used culinary fat, VCO integrates naturally into daily diets, supporting its relevance as a practical nutritional factor influencing neurocognitive function. Collectively, available findings highlight VCO as a promising dietary component with relevance for supporting brain health and cognitive resilience through complementary metabolic and neuroprotective mechanisms.

#### 4.7.2 Effects on brain health and cognitive function

VCO may supports cognitive function and brain health primarily via its MCTs, which provide ketone bodies as alternative neuronal energy substrates. These effects are closely related to reductions in oxidative stress and inflammation, key contributors to cognitive decline. While MCTs are also present in other lipid sources such as palm kernel oil and purified MCT oil, VCO is distinguished by its unique composition, particularly its high LA (C12) content and the presence of bioactive compounds such as polyphenols and tocopherols. Coconut oil is recognized as the richest natural dietary source of LA, accounting for approximately 45-50% of its total fatty acids, which are substantially higher than in other dietary fats (Hewlings, 2020). Unlike refined MCT oils, which are composed almost exclusively of caprylic (C8) and capric (C10) acids, VCO contains a broader spectrum of fatty acids, with LA being the dominant component, along with minor bioactive constituents (McKenzie et al., 2021). Furthermore, emerging evidence suggests that whole coconut oil exhibits biological effects that differ from isolated MCTs, likely due to synergistic interactions between MCFAs and accompanying bioactive compounds (Gao et al., 2022). This complex lipid matrix may contribute to its neuroprotective and antioxidant effects beyond simple ketone body production.

Moreover, human studies provide preliminary evidence. In adults with metabolic syndrome, daily consumption of 30 mL VCO for four weeks improved oxidative stress biomarkers, total antioxidant capacity, and insulin resistance indicators (Mansouri et al., 2024). Serum BDNF increased within the VCO group, although not significantly versus controls. Similarly, in a 6-month study of individuals with mild-to-moderate AD, APOE ε4 carriers receiving VCO supplementation demonstrated improved cognitive performance as measured by MMSE scores (Fernando et al., 2023).

Preclinical studies verify these findings. In mice exposed to chronic unpredictable mild stress, VCO improved stress-related cognitive impairments, enhanced hippocampal antioxidant activity, and upregulated GABAA and mGluR1a receptor expression (Edem et al., 2022). In obese and healthy rats, E-VCO reduced energy intake and weight gain, exhibited antidepressant-like behaviors, protected hippocampal neurons, and modulated gut-brain communication by shifting macrophage polarization from pro-inflammatory (M1) to anti-inflammatory (M2) (Araújo de Vasconcelos et al., 2023).

**Fig. 8:**
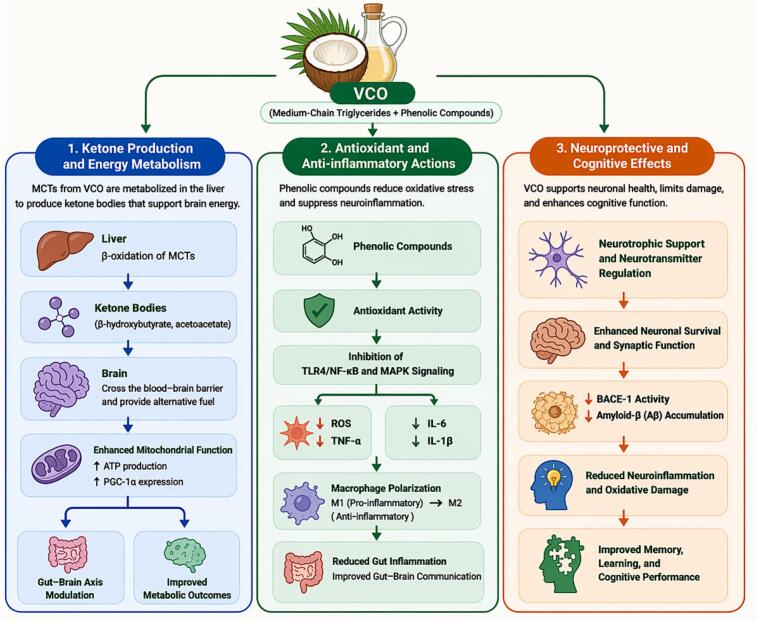
Schematic representation of the proposed mechanisms by which VCO supports brain health and cognitive function. MCTs are metabolized in the liver via β-oxidation to generate ketone bodies (β-hydroxybutyrate and acetoacetate), which cross the blood–brain barrier and serve as alternative energy substrates, enhancing mitochondrial ATP production and PGC-1α expression. Concurrently, VCO phenolic compounds exert antioxidant and anti-inflammatory effects by reducing oxidative stress (ROS decreased) and suppressing TLR4/NF-κB and MAPK signaling pathways, leading to decreased pro-inflammatory mediators (TNF-α, IL-6, IL-1β) and promoting macrophage polarization (M1 → M2). These coordinated mechanisms improve gut–brain axis communication, reduce neuroinflammation and oxidative damage, and contribute to enhanced memory, learning, and cognitive performance.

Additional animal studies highlight age- and hormone-related cognitive benefits. Aging female rats receiving VCO supplementation showed improved learning, memory retention, and task performance in Y-maze assessments (Young et al., 2021). VCO also slowed cortical spreading depression propagation, a marker of brain excitability, without affecting lipid peroxidation (Alves et al., 2021). Fig. 8 represents the proposed mechanistic pathway through which VCO enhances brain health and cognitive function by providing ketone bodies as alternative neuronal energy substrates, reducing oxidative stress and inflammation, upregulating neurotrophic and synaptic signaling pathways, modulating the gut-brain axis, and ultimately improving memory, learning, and neuroprotection.

Collectively, findings from preclinical and early clinical investigations demonstrate that VCO influences key aspects of brain health, including cognitive performance, oxidative balance, and neuronal signaling. Experimental studies provide strong mechanistic evidence linking VCO and its MCFAs to antioxidant protection, modulation of neuronal signaling pathways, and preservation of cellular integrity within the nervous system. Human studies further indicate that VCO intake is associated with improvements in selected cognitive and antioxidant-related outcomes, particularly when incorporated into habitual dietary patterns. These observations highlight the relevance of VCO as a practical dietary component capable of supporting neurocognitive function through complementary metabolic and neuroprotective mechanisms. Taken together, the available evidence establishes a solid foundation supporting the role of VCO in brain health and cognitive resilience, underscoring its value as a functional dietary fat with meaningful implications for neurological well-being.

#### 4.7.3 Effects on Alzheimer’s disease (AD)

VCO may mitigate AD-related neurodegeneration by providing ketone bodies as alternative neuronal energy substrates (Asih et al., 2014; Ota et al., 2019). Animal models indicate that VCO supplementation reduces amyloid-β accumulation, hyperphosphorylated tau, oxidative stress, and neuroinflammation while improving cognitive function. For example, dietary VCO (8-10%) in Wistar rats with Aβ-induced AD restored hematological and biochemical parameters, enhanced hippocampal antioxidant enzymes, reduced nitric oxide and cholinesterase activity, and suppressed TNF-α and iNOS expression (Mirzaei, Khazaei, Komaki, Amiri and Jalili, 2019a, Mirzaei, Khazaei, Komaki, Amiri and Jalili, 2019b). Oral administration of 5 mL VCO for six weeks similarly improved learning, memory, and antioxidant levels in AlCl3-induced AD rats (Alnefaie & Bawazir, 2024). VCO-fortified dairy formulas improved ketone production, reduced lipid peroxidation, activated Nrf2/HO-1 signaling, and decreased matrix metalloproteinase activity (Khalil et al., 2020).

Cellular studies further support these mechanisms. In SH-SY5Y cells exposed to aluminum, VCO reduced ROS and lipid peroxidation, improved dopamine and ACh levels, and lowered hyperphosphorylated tau and amyloid-β (Demirel et al., 2024). Phenolic compounds in VCO also contribute to reducing amyloid plaque formation and oxidative damage (Al-Matrafi, 2021). The effects of VCO on AD are summarized in Table 7.

**Table 7:** Studies investigating the potential effects of VCO on Alzheimer’s Disease (AD).

References	Study Type	Sample Type	Effects of VCO on Alzheimer's Disease	Key Findings
(Fernando et al., 2023, Fernando et al., 2023)	*In vivo* (human)	Elderly individuals / mild-to-moderate AD patients	30 mL/day VCO vs canola oil, double-blind trial, 24 weeks	No significant group differences in overall cognitive or metabolic markers; however, MMSE scores improved specifically in APOE ε4 carriers, with no significant lipid profile changes
(Mirzaei et al., 2019a)	*In vivo* (animal)	Wistar rats	VCO treatment in experimentally induced AD model	Normalized hematological parameters and oxidative stress markers in AD rats
(Mirzaei et al., 2019b)	*In vivo* (animal)	Wistar rats	VCO intervention in AD rat model	Improved antioxidant enzyme activity, reduced TNF-α and nitric oxide levels, decreased tau phosphorylation and amyloid plaque formation
(Alnefaie & Bawazir, 2024)	*In vivo* (animal)	Wistar rats	VCO treatment against AlCl3-induced neurodegeneration	Enhanced memory function and reduced AlCl3-induced oxidative stress and neuronal damage
(Alghamdi, 2018)	*In vivo* (animal)	Rats	VCO neuroprotective effects	Preserved cortical and hippocampal structural integrity, prevented neurodegeneration
(Khalil et al., 2020)	*In vivo* (animal)	Wistar rats	VCO-enriched formula intervention	Reduced oxidative stress, improved cognitive function, restored brain structural integrity, and regulated matrix metalloproteinases (MMP-2 and MMP-9)

Collectively, preclinical and cellular studies demonstrate that VCO engages multiple pathways relevant to AD, including enhanced ketone availability, regulation of oxidative balance, modulation of inflammatory signaling, and influence on amyloid- and tau-associated processes. Across animal and in vitro models, VCO-based interventions have consistently been associated with improved antioxidant status, modulation of amyloid-β accumulation, and enhanced cognitive performance, providing a strong biological rationale for its relevance in neurodegenerative research. Variations in experimental approaches, including differences in formulation (whole VCO, phenolic-rich fractions, or MCT-enriched preparations), dosage, and duration, highlight the contribution of distinct bioactive components of VCO to these neuroprotective effects.

Human studies further indicate that VCO-based dietary interventions are associated with favorable metabolic and cognitive responses when incorporated into broader dietary patterns. These observations underscore the translational relevance of VCO as a nutritional factor capable of supporting brain metabolism and cognitive function in AD-related contexts. Taken together, the available evidence establishes a compelling mechanistic and translational foundation supporting the neuroprotective potential of VCO in AD. These findings highlight VCO as a functional dietary component with meaningful implications for supporting cognitive health and neuronal resilience in populations affected by neurodegenerative conditions.

### 4.8 Bone loss prevention effects

Oxidative stress and free radicals are key contributors to the development of osteoporosis, making antioxidant strategies important for prevention and treatment. VCO rich in MCFAs and polyphenolic compounds have bone-protective effects due to its antioxidant and anti-inflammatory properties. Several *in vivo* studies using ovariectomized (OVX) rat models, which mimic postmenopausal osteoporosis, have highlighted the potential that VCO may preserve bone structure and function. For example, Hayatullina et al. (2012) examined the effects of dietary supplementation with 8% VCO over six weeks in three-month-old female rats (n = 32), assigned to four groups: baseline, sham-operated, OVX control, and OVX with VCO supplementation. Rats in the VCO-supplemented group exhibited significantly better bone volume, increased trabecular number, and reduced trabecular separation compared to the OVX control group, indicating that VCO helped preserve bone microarchitecture. Similarly, Abujazia et al. (2012) demonstrated that VCO reduced oxidative stress in bone tissue by significantly decreasing MDA levels and increasing the activities of GPx and SOD in OVX rats (n = 32), treated with 8% dietary VCO for six weeks. These findings suggest that VCO may not only improves bone morphology but also enhances antioxidant defenses in estrogen-deficient conditions.

Furthermore, combining VCO with tocotrienol-rich fraction (TRF) appears to yield synergistic effects in improving bone strength and reducing bone resorption in OVX models. In a 24-week study by Malik et al. (2019), 36 female Sprague-Dawley rats were assigned to six groups: sham, OVX control, OVX + Premarin (64.5 μg/kg), OVX + VCO (4.29 ml/kg), OVX + TRF (30 mg/kg), and OVX + VCO + TRF (4.29 ml/kg + 30 mg/kg). The combination therapy significantly reduced serum levels of C-terminal telopeptide of Type I collagen (CTX-1), a marker of bone resorption, and enhanced bone formation rates in dynamic histomorphometry analyses. In a related study by Malik et al. (2020), OVX rats were fed a high-fat diet and heated palm oil for 24 weeks and were similarly treated with VCO, TRF, or both. Biomechanical testing showed that the combined VCO and TRF group (1.43 ml/kg + 30 mg/kg) exhibited a significantly higher young’s modulus compared to single-treatment groups, indicating superior intrinsic bone strength. These findings highlight the added benefits of co-supplementation, particularly in lipid-induced bone deterioration models.

Additionally, in another relevant study addressing obesity-associated bone loss, Zicker et al. (2022) explored the effects of VCO in a diet-induced obesity mouse model. Male BALB/c mice were initially fed a high-refined carbohydrate diet for eight weeks to induce obesity and then supplemented with varying doses of VCO for four weeks. The study reported that VCO improved trabecular bone microarchitecture in the tibia, lumbar vertebrae, femur, and alveolar bone, and enhanced alkaline phosphatase (Alp) expression. Moreover, VCO supplementation significantly reduced the Rankl/Opg ratio, a key regulator of bone remodeling, suggesting a beneficial modulation of inflammatory and osteogenic pathways in obesity-related osteopenia.

Overall, VCO supports bone health through its ability to reduce oxidative stress, modulate bone remodeling processes, and preserve bone microarchitecture, particularly under conditions such as estrogen deficiency or metabolic challenge. Preclinical and mechanistic studies consistently demonstrate these effects under controlled dietary conditions, highlighting the contribution of VCO’s bioactive components to skeletal integrity and bone metabolic balance. Human studies further indicate that incorporation of VCO into balanced dietary patterns is associated with favorable modulation of bone-related markers. These observations support the role of moderate VCO intake, particularly as a partial replacement for other dietary fats, in promoting bone health and maintaining skeletal resilience. Collectively, available evidence underscores the relevance of VCO as a functional dietary component with meaningful potential for supporting bone health within realistic consumption patterns.

### 4.9 Hepatoprotective effects

VCO exhibits significant hepatoprotective effects, primarily attributed to its rich content of MCFAs and potent antioxidants such as polyphenols. These bioactive compounds help mitigate oxidative stress and inflammation, thereby protecting the liver from damage induced by toxins, drugs, HFDs, and heavy metals. In an *in vivo* study by Eleazu et al. (2019), 24 male albino rats were divided into four groups (n = 6), including a diabetic control and a diabetic group treated with 5 mL/kg VCO daily. VCO supplementation significantly reduced higher liver enzymes (ALT, AST, ALP) and improved antioxidant defenses, including CAT, GPx, and GSH levels, while reducing MDA in liver tissues. Similarly, in a 16-week study by de Vasconcelos et al. (2022), 32 male Wistar rats were divided into four groups (n = 8) to evaluate the effects of 3000 mg/kg/day of encapsulated VCO (E-VCO) on obesity-induced liver dysfunction. E-VCO supplementation significantly enhanced the activity of hepatic antioxidants (SOD and GPx), reduced hepatic fat accumulation, and lowered hepatic triglyceride and cholesterol levels. Moreover, it promoted fecal cholesterol excretion and improved liver and adipose tissue histology in obese rats, demonstrating its ability to regulate lipid metabolism under metabolic stress.

Further supporting the antioxidant potential of VCO, Sinaga et al. (2020) conducted a 28-day *in vivo* experiment in 24 male rats (n = 6), where oxidative stress was induced by forced maximal swimming. Rats treated with VCO at doses of 1, 2, and 4 mL/200 g body weight via oral gavage exhibited significantly increased GPx activity and swimming endurance, along with decreased levels of MDA, ALT, and AST. Similarly, in a study by Famurewa et al. (2021), rats pretreated with polyphenols isolated from VCO at doses of 10, 20, or 50 mg/kg for seven weeks (including two weeks before cadmium exposure) showed improved antioxidant status, demonstrated by elevated SOD, CAT, GPx, and GSH, and reduced MDA levels. Histopathological analysis confirmed reduced liver damage. Lokesha et al. (2021) also reported hepatoprotection against cadmium toxicity in 24 rats (n = 6), where daily oral VCO administration at 2 mL/kg for 30 days significantly decreased serum SGPT and SGOT levels and minimized histological liver damage compared to the cadmium-only group. These findings consistently demonstrate that VCO and its derived polyphenols improve liver antioxidant capacity and respond oxidative liver injury induced by physical stress and heavy metals.

In models of drug-induced hepatotoxicity, Abdel et al. (2023) evaluated the protective effect of VCO (10 mL/kg/day for 7 days) against doxorubicin-induced liver injury in rats (n = 6 per group). VCO pre-treatment restored antioxidant enzyme levels (SOD, CAT, GSH), reduced MDA and serum liver enzymes (ALT, AST), and modulated apoptotic and inflammatory markers, although it failed to suppress NF-κB and pro-inflammatory cytokines (TNF-α, IL-6), possibly due to its saturated fat content. Similarly, in a study by Abbasi et al. (2021), VCO (6.7 mL/kg/day for 15 days) was compared with Neem extract in 60 Wistar and Sprague-Dawley rats (15 per group) with acetaminophen-induced liver injury. Both treatments significantly lowered serum ALT, AST, and ALP levels compared to the acetaminophen-only group, and VCO-treated rats showed enzyme levels comparable to healthy controls, confirming its protective role against drug-induced hepatotoxicity.

**Fig. 9:**
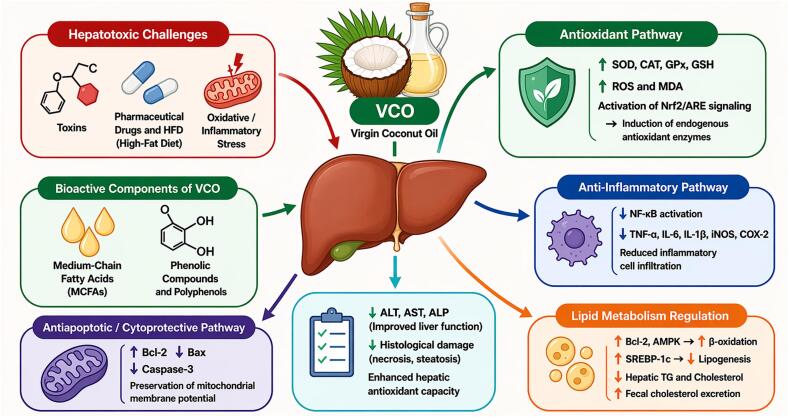
Schematic overview of the hepatoprotective mechanisms of VCO. VCO bioactive components (MCFAs and phenolic compounds) attenuate hepatotoxic stress by enhancing antioxidant defenses via the Nrf2/ARE pathway (↑ SOD, CAT, GPx, GSH; ↓ ROS, MDA) and suppressing inflammation through inhibition of NF-κB signaling (↓ TNF-α, IL-6, IL-1β, iNOS, COX-2). VCO also regulates lipid metabolism (↑ β-oxidation via AMPK; ↓ SREBP-1c–mediated lipogenesis) and exerts antiapoptotic effects (↑ Bcl-2, ↓ Bax, ↓ caspase-3), collectively improving liver function and reducing tissue damage.

Moreover, VCO has demonstrated benefits in lipid regulation and antioxidant defense in dietary liver injury models. Arsang et al. (2020) investigated the effects of VCO in a high-fat diet-induced fatty liver model in 30 male rats (n = 5 per group). Supplementation with 8% and 10% VCO mixed into the diet significantly reduced serum triglycerides (from 159 ± 11.5 to 104.5 ± 9.1 and 97.5 ± 8.2, respectively) and cholesterol, while enhancing hepatic antioxidant status. These findings support the role of VCO in ameliorating hepatic lipid accumulation and oxidative stress associated with diet-induced hepatic steatosis. Additionally, an *in vivo* study by Allam et al. (2024) used a carbon tetrachloride (CCl₄)-induced acute liver failure model in adult male albino rats to examine the hepatoprotective effects of conventional and nanoparticle formulations of VCO and GSH. Over seven days of treatment, the combined VCO-GSH nanoparticle formulation significantly reduced serum ALT and AST levels, oxidative stress (MDA), and pro-inflammatory cytokines (TNF-α, IL-1β). Histopathological and immunohistochemical analyses showed reduced hepatocellular necrosis and NF-κB expression, indicating the enhanced therapeutic potential of nano-formulated VCO in acute liver injury. The proposed molecular mechanisms through which VCO exerts its hepatoprotective effects are illustrated in Fig. 9, summarizing its roles in modulating oxidative stress, inflammation, lipid metabolism, apoptosis, and mitochondrial integrity.

Collectively, preclinical studies demonstrate that VCO supports liver health through improvements in liver function biomarkers, enhancement of antioxidant defenses, reduction of hepatic lipid accumulation, and modulation of inflammatory pathways across diverse models of hepatic challenge, including metabolic stress, toxin exposure, drug-induced injury, and heavy metal exposure. These hepatoprotective effects are primarily attributed to the MCFAs and polyphenolic compounds present in VCO, which act synergistically to promote hepatic resilience and metabolic balance. Human studies further indicate that incorporation of VCO into balanced dietary patterns is associated with favorable modulation of liver-related biomarkers, particularly when used as a partial replacement for other dietary fats. Together, these findings highlight VCO as a functional dietary component with meaningful relevance for supporting liver health and maintaining hepatic homeostasis within realistic consumption patterns.

### 4.10 Dosage and practical recommendations of VCO

Generally, a daily dose of 1-2 tablespoons (15-30 mL) of VCO is commonly recommended to balance potential physiological benefits with overall dietary energy intake. Each tablespoon provides approximately 120 kcal, and gradual introduction is typically advised to allow adaptation, with intake tailored to individual health goals and responses (Liau et al., 2011). Across multiple clinical studies, a daily intake of 15-30 mL of VCO has been applied in adults aged 20-50 years, with reported benefits including improvements in HDL cholesterol, reductions in triglycerides and fasting blood glucose, and enhancement of oxidative stress markers (Mansouri et al., 2024; Rahmawati et al., 2023). In older adults with mild-to-moderate AD, comparable dosages have been evaluated over extended intervention periods, focusing on condition-specific metabolic and cognitive outcomes (Fernando et al., 2023). Furthermore, evidence in younger populations remains limited; however, a controlled study in males aged 19-40 years demonstrated reduced hunger and appetite following VCO consumption compared with olive oil, indicating potential relevance for satiety regulation and body weight management (Metin et al., 2022).

VCO has also been investigated as a dietary adjunct in clinical settings. In patients with mild-to-moderate COVID-19, supplementation alongside standard care was associated with faster symptom resolution and earlier normalization of inflammatory markers, suggesting potential immunometabolic support (Angeles-Agdeppa et al., 2024). Moreover, in healthy adults, daily consumption of 30 mL of VCO has been shown to increase HDL cholesterol, supporting its role in lipid modulation within the general population (Chinwong et al., 2017).

Overall, available clinical evidence indicates that moderate VCO intake (15-30 mL/day) may support favorable outcomes related to lipid metabolism, glycemic control, oxidative balance, appetite regulation, and selected condition-specific health endpoints. Practical incorporation of VCO should consider its energy density and integration within habitual dietary patterns.

## 5 Applications of VCO

VCO has a wide range of applications across various sectors, including food, nanotechnology, and therapeutic uses. Several studies have highlighted its potential in these areas which are given below:

### 5.1 Functional foods

The growing demand for safe, natural, and health-promoting products has driven rapid advancements in functional food development, focusing on providing healthier dietary options. VCO, due to its high concentration of MCFAs is being recognized as a valuable alternative with potential applications as both a therapeutic agent and a functional food ingredient. Current research efforts are focused on expanding its utility in various food products, which will be further elaborated below:

#### 5.1.1 Low-calorie fat substitute

Among the growing health concerns consumers are driving a shift in dairy products like mayonnaise, cheese analogues, and ice cream, with high-fat ingredients increasingly substituted by vegetable oils or fats, including VCO (Aini et al., 2020; Choo et al., 2010; Muhialdin et al., 2019). VCO has been used as a substitute for milk fat in ice cream resulting in desirable appearance, texture, and flavor (Choo et al., 2010). Similarly, spreads formulated with VCO, trans fat free alternatives, spice mixtures, and coconut residue have shown outstanding spreadability, stability, and sensory acceptability (Hitlamani et al., 2023). Additionally, a cheddar cheese analogue made from corn milk incorporating (15-25% w/w, VCO) along with the Tween 80 as emulsifier exhibited favorable color, aroma, taste, and texture (Aini et al., 2020). Replacing soybean oil with VCO in mayonnaise formulations enhanced the product's quality while maintaining its organoleptic properties (Muhialdin et al., 2019). Additionally, in a study conducted by Mohammed et al. (2022), VCO was used to develop an emulsion that closely simulated the properties of a low-fat substitute. In this formulation, emulsified VCO functioned as an egg substitute in mayonnaise enhancing its texture, stability, and viscosity. The formation of egg free mayonnaise with VCO is becoming increasingly relevant for vegan and health-conscious consumers. However, additional research is needed to evaluate the sensory qualities and storage stability of these VCO based, egg free mayonnaise products. Notably, VCO has been shown to positively impact lipid profiles by improving the LDL-to-HDL cholesterol ratio and reducing overall cholesterol, phospholipids, and triglycerides in the bloodstream (Aini et al., 2020), It shows potential as a low-fat alternative in a range of dairy products.

Importantly, the use of VCO in these formulations not only replaces high-calorie or trans-fat sources but also influences its inherent health-promoting properties, such as improving lipid metabolism and enhancing antioxidant defense. By incorporating VCO into low-fat and egg-free emulsions, these products can potentially deliver cardiovascular and metabolic benefits in addition to desirable sensory attributes. This demonstrates a direct translation of VCO’s functional properties particularly its favorable impact on lipid profiles into practical food applications that align with health-driven consumer demands.

#### 5.1.2 VCO in beverages

VCO has garnered increasing interest as a functional ingredient in a variety of beverage formulations due to its unique health benefits and sensory properties. As a rich source of MCFAs, VCO is easily digestible and provides a quick source of energy, making it a valuable addition to functional drinks, smoothies, and coffee (Kumar et al., 2024; Pandiselvam et al., 2024). The creamy texture and mild coconut flavor of VCO enhance the taste and mouthfeel of beverages, while its inherent antimicrobial and antioxidant properties contribute to improved shelf stability and align well with the growing consumer demand for nutritious, plant-based products (Stefanidis et al., 2023). In a study by Bhattacharjee (2021) developed an antioxidant-rich ready-to-serve (RTS) beverage using irradiated VCO combined with green tea extract. The irradiated VCO extended the shelf life of the beverage to 13 days at 4°C, and further microencapsulation significantly increased its dry form stability by 29 times, indicating the application of VCO in health beverages.

Additionally, research on VCO-based emulsions has also explored methods to enhance beverage stability. Yani et al. (2018) assessed VCO emulsions (5%-30%) stabilized with soy lecithin, evaluating viscosity, pH, and cream formation. The 20% emulsion showed the highest stability, though challenges with cream separation highlighted the need for further optimization. Additionally, a review by Jayasekara and Gunathilake (2007) discussed the potential of utilizing defatted residues from VCO extraction as high-value protein and fiber-rich products and the possibility of producing beverages from both tender and mature coconut water, adding value to the VCO production process.

Despite the potential applications of VCO in the beverage industry, the existing body of research is relatively limited, and further studies are required to fully explore its potential. A recent systematic review by Parisi et al. (2024) emphasized the importance of packaging, durability, and sustainability in enhancing the value of coconut-based beverages. Factors like recyclable packaging materials, varied size options, and certified organic and fair-trade practices could play a significant role in boosting consumer appeal and supporting the growth of the coconut industry. Overall, while preliminary research suggests that VCO may enhance the nutritional and functional profile of beverages, more comprehensive studies are needed to overcome current formulation challenges and unlock its full potential for innovative health drinks.

To conclude, VCO-based beverages not only offer improved sensory and nutritional qualities but also leverage VCO’s bioactive components to enhance health outcomes. Its MCFAs and antioxidant compounds support energy metabolism, cardiometabolic health, and oxidative stress modulation, while antimicrobial properties improve shelf stability. Formulation strategies such as emulsions, nano-emulsions, and microencapsulation further protect these bioactive compounds, enhance bioavailability, and maintain functional efficacy in beverage matrices. Additionally, utilizing VCO-derived residues and coconut water can increase protein and fiber content, providing added nutritional benefits. These approaches demonstrate a clear “from properties to applications” framework, explaining mechanistic health effects into practical beverage products. Future research should optimize formulation parameters, processing methods, and packaging strategies to overcome stability challenges and maximize functional, sensory, and sustainable attributes. Overall, VCO-enriched beverages represent a potential avenue for developing innovative, health-promoting drinks that directly connect VCO’s inherent biological properties to consumer-ready applications.

#### 5.1.3 VCO in baked goods

VCO is increasingly recognized as a valuable ingredient in the baking industry, serving as a healthier alternative to traditional fats like butter and shortening due to its high content of MCFAs. The MCFAs in VCO improve crumb softness and add a mild coconut flavor, enhancing the sensory qualities of baked products (Varghese et al., 2024). Additionally, VCO's stability at high temperatures makes it particularly suitable for various baking processes, as it improves moisture retention and extends the shelf life of baked goods (Gani & Benjakul, 2020). The antimicrobial and antioxidant properties of VCO further reduce spoilage, making it an attractive choice for functional and health-focused bakery applications. Moreover, research by Rosida et al. (2024) demonstrated the use of VCO in kimpul-based cookies, where replacing traditional margarine with 40% VCO significantly improved the functional, physicochemical, and sensory characteristics of the cookies, indicating its potential in producing high-quality baked products.

Moreover, studies have also explored the utilization of VCO byproducts in baking applications, suggesting their nutritional and functional benefits. Beegum et al. (2017) investigated the substitution of refined wheat flour with VCO cake (0-50 g/100 g) in muffins, finding that muffins with 40 g VCO cake exhibited high acceptability and were enriched with protein, fat, fiber, and minerals. Similarly, Srivastava et al. (2018) reported the use of residual virgin coconut meal (VCM) a byproduct of VCO extraction in baked goods such as cakes and biscuits. Incorporating VCM not only improved the nutritional profile of these products by increasing protein and fiber content but also enhanced the batter viscosity and modified the gelatinization process (Srivastava & Semwal, 2015). Furthermore, Fonseca et al. (2023) optimized the incorporation of coconut flour, another byproduct of VCO production, in snack formulations. The resulting products demonstrated improved physicochemical properties and higher protein, fat, and ash content, highlighting the potential of coconut flour in developing healthier baked snacks. Additionally, Srivastava (2010) found that replacing refined wheat flour with 5-25% VCM in biscuits increased redness and yellowness while enhancing dough hardness and reducing stickiness, highlighting the positive textural and sensory changes associated with the use of VCM.

**Fig. 10:**
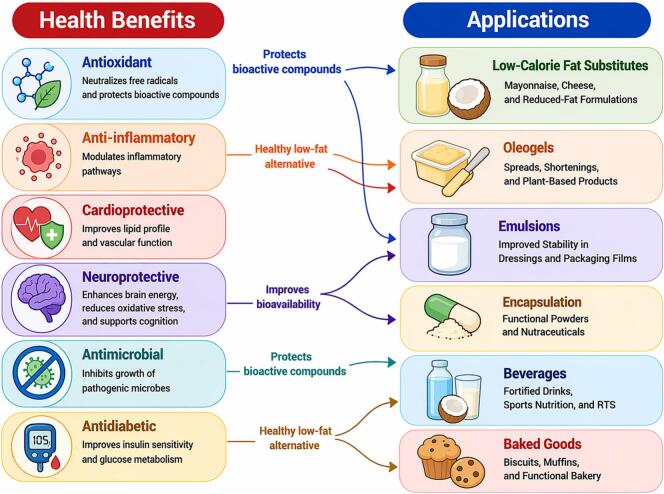
Health benefits and translational applications of VCO, highlighting its major bio functional properties and potential uses in food and nutraceutical products.

Incorporating VCO into baked goods not only improves sensory attributes such as texture, flavor, and moisture retention but also enhances the nutritional and functional value of these products. Its MCFAs and antioxidant compounds contribute to energy metabolism, oxidative stress reduction, and microbial stability, while its thermal stability ensures the preservation of these bioactive compounds during baking. The use of VCO-based byproducts, such as coconut flour, VCO cake, and residual VCM, further enriches protein, fiber, and mineral content, linking VCO’s health-promoting properties to practical bakery applications. These formulation strategies protect bioactive components, improve functional efficacy, and align with health-focused and low-fat product development. Future studies should optimize incorporation levels, processing conditions, and storage stability to maximize both health benefits and sensory quality. As illustrated in Fig. 10, the diverse health-promoting properties of VCO have enabled its translation into a wide range of food and nutraceutical applications, including its use as a low-calorie fat substitute, oleogel component, emulsifier, encapsulating agent, and functional ingredient in beverages and baked products. To conclude, VCO-enriched baked products represent a viable route for delivering functional ingredients while promoting healthier and more sustainable bakery formulations.

### 5.2 Delivery systems

#### 5.2.1 VCOs encapsulation

VCO is rich in bioactive compounds including phenolic compounds such as VA, gallic acid, protocatechuic acid, SA, CA, FA, and P-CA. However, these compounds are susceptible to degradation when exposed to temperature variations, moisture, oxygen, and light (Satheesh & Prasad, 2012). Encapsulation techniques provide a protective barrier for these bioactive compounds, improving their oxidative stability, dispersibility, and bioavailability (Priyanto et al., 2022). Commonly used encapsulation methods include spray-drying (Nurhadi et al., 2020; Øye et al., 2023), freeze-drying (Mohammed et al., 2020), fluidized bed coating (Reineccius et al., 2022; Sousa et al., 2022), and coacervation phase-separation followed by freeze drying (Wong & Hartina, 2014). Spray drying is commonly used to transform liquid foods into powdered forms due to its benefits, including rapid evaporation, drying at wet bulb temperatures, cost-effectiveness and minimal contact time with hot air (Mohammed et al., 2020). In the encapsulation of VCO, the oil is emulsified with wall materials and then spray dried to produce powder.

Furthermore, VCO encapsulated with sodium caseinate and maltodextrin has demonstrated a high encapsulation efficiency of 80.51%, as well as significant oxidative stability and antioxidant activity (Hee et al., 2017). Additionally, Nurhadi et al. (2022) developed a Pickering emulsion that was spray dried to produce encapsulated VCO powders which can serve as a non-dairy creamer in food formulations. Although spray-drying uses lower temperatures, the exposure of dried powders to hot air during their passage to the cyclone separator can still degrade heat sensitive VCO. Furthermore, the process often results in a wide range of particle sizes.

Moreover, Mohammed et al. (2024) produced a non-dairy cream powder from VCO through a combination of fluidized bed coating techniques and spray drying. In fluidized bed coating an emulsion is applied to a fluidized bed and dried with an air stream, while maintaining strict control over humidity and temperature conditions (Sousa et al., 2022). Encapsulated VCO powders produced through this method showed improved oxidative stability, reconstitution properties, and flowability (Mohammed et al., 2020). Fluidized bed coating offers the benefits of high thermal efficiency, low operational costs, and temperature control though it is time consuming (Sousa et al., 2022).

The Supercritical Fluid Extraction of Emulsions (SFEE) method is an innovative and scalable technique for encapsulating bioactive compounds (Cerro et al., 2024). This process utilizes supercritical fluids to extract the organic phase containing wall materials and encapsulated bioactive microparticles or microcapsules free from solvent traces (Klettenhammer et al., 2022). These microspheres demonstrate high encapsulation efficiency and a significant bioactive ingredient load (Hee et al., 2017; Klettenhammer et al., 2022). In this approach, emulsion based on VCO is introduced into a precipitation chamber where it is solubilized using supercritical CO2. The subsequent reduction in pressure results in the formation of encapsulated VCO powders (Hee et al., 2017). Furthermore, Hee et al. (2017) used supercritical CO2 to encapsulate VCO from an oil-in-water emulsion achieving an encapsulation efficiency of 80.2% under mild-operating conditions (16 MPa and 40°C). This process provides advantages such as accurate control over morphology and particle size, as well as improved solubility and stability in aqueous systems (Cerro et al., 2024).

However, VCO produced via fermentation has been shown to exhibit anti-fungal activity against *candida* species and other fungi (Ogbolu et al., 2007). Furthermore, Hariyadi et al. (2022) hydrolyzed VCO using *R miehei* lipase and encapsulated it with sodium-alginate through extrusion, forming VCO microspheres. These microspheres exhibited significant anti-bacterial and anti-fungal properties. Encapsulation enables the handling of VCO in the form of free-flowing powders, prolonging shelf-life during storage and enhancing its convenience in functional food applications.

Encapsulation techniques serve not only to protect VCO’s bioactive compounds from environmental degradation but also to enhance their stability, bioavailability, and controlled release in functional foods and nutraceutical formulations. By maintaining the integrity and efficiency of these compounds, encapsulation ensures that the health-promoting properties of VCO are effectively delivered in practical applications, including vegan, low-fat, or fortified products. These approaches demonstrate the capacity to explain VCO’s molecular and functional attributes into tangible industrial solutions, bridging experimental evidence with real-world product development.

#### 5.2.2 Oleogels formulated with VCO

Oleogels are semi solid fats formed by trapping liquid vegetable oils within a thermo reversible three-dimensional gel network using oleogelators or structuring agents. The final properties of oleogels such as texture, opacity, oil binding, melting point, and capacity are determined by the characteristics of the oleogelators, which include concentration, molecular weight, crystallinity, hydrophobic to hydrophilic balance, and glass transition temperature. Furthermore, the properties of the resulting oleogels are influenced by processing methods, process parameters, and the degree of unsaturation in the liquid oil phase (Manzoor et al., 2022). Presently, research efforts are directed toward formulating oleogels for the encapsulation and delivery of lipophilic bioactive compounds in applications related to nutraceuticals, food, and pharmaceuticals (Manzoor et al., 2022; Pușcaș et al., 2020).

Oleogels with VCO as the oil phase are being developed through a FA crystallization mechanism (Thomas et al., 2023). In this process, oleogelators are dispersed within the oil phase (VCO), followed by sequential heating and cooling. During the cooling phase these oleogelators reorganize into a crystalline or polymeric network forming the oleogel (Manzoor et al., 2022; Valoppi et al., 2020). Table 8 presents the structural components utilized in oleogels based on VCO and their corresponding applications in food. Oleogels formed from VCO and stearic acid exhibit textural properties like firmness, stickiness, spreadability, and adhesion (Thomas et al., 2023). These oleogels demonstrate potential as alternatives to solid-fats, including trans and saturated fats, in various food formulations such as spreads, shortenings, baked goods, confectionery items, meat products, dairy products, and fried snacks (Thomas et al., 2023). Moreover, oleogels formulated with ethyl cellulose and VCO exhibited a significant oil-binding capacity and excellent thermal stability. Additionally, edible oleogels composed of beeswax and VCO were shown to improve the stability of VCO's bioactive compounds by protecting them from oxidation during storage (Gengatharan et al., 2023). Furthermore, oleogels have potential applications in the development of edible oleofilms and 3D-printed food products (Thomas et al., 2023).

**Table 8:** Overview of different products derived from VCO and their corresponding applications in food.

References	Structural components	System/ product	Method	Food applications
(Dhulipalla et al., 2023)	40 wt% stearic acid; 20 wt% VCO; 40 wt% lycopene (tomato)	VCO-based oleogel	FA crystallization	Lycopene delivery / encapsulation
(Thomas et al., 2023)	Stearic acid + VCO (2-10 wt%)	VCO-based oleogel	FA crystallization	Fat replacer in food products
(Silva et al., 2008)	Ethyl cellulose (5-15 wt%) + VCO (85-95 wt%)	VCO-based oleogel	FA crystallization	Bioactive compound delivery
(Tripathi et al., 2017)	Candelilla wax (6-12 wt%) or beeswax (6-12 wt%) + VCO (88-94 wt%)	VCO-based oleogel	FA crystallization	Fat replacer in food products
(Pușcaș et al., 2020)	Beeswax (7.7-15.5 wt%) + VCO (84.5-92.4 wt%)	VCO-based oleogel	FA crystallization	Shortening replacer in cookies
(Suraweera et al., 2014)	Tween 80 (30.53 wt%); water (45.87 wt%); VCO (23.6 wt%); ketoprofen (2.5 wt%)	VCO-based creamy nanoemulsion	Homogenization (10,000 rpm/5 min)	Drug delivery
(Sanjeewani & Sakeena, 2013)	Tween 80 (32 wt%); water (36 wt%); VCO (32 wt%)	O/W nanoemulsion	Homogenization (10,000 rpm/5 min)	Drug delivery
(Gani & Benjakul, 2018)	Sodium caseinate (3 wt%); water (95 wt%); VCO (2 wt%)	VCO-based nanoemulsion	Ultrasonication + homogenization (11,000 rpm/15 min)	Uniform fat/oil distribution in croaker surimi gel
(Gani & Benjakul, 2018)	α-Tocopherol; epigallocatechin gallate; VCO; β-glucan powder	VCO-based nanoemulsion	Ultrasonication (5 min; 750 W; 20 kHz ± 50 Hz; 60% amplitude)	Reduced microbial load and improved oxidative stability in croaker surimi paste
(Pengon et al., 2018)	Water; hydrogenated castor oil (5 wt%)	O/W nanoemulsion	Homogenization (10,800 rpm/5 min)	Improved oxidative stability and reduced microbial load in croaker surimi paste
(Liu et al., 2013)	Whey protein isolate; green tea seed oil; tea saponin; soy lecithin (0.5-10 wt%); VCO (10 wt%)	O/W nanoemulsion	Homogenization (20,000 rpm/2 min) + ultrasonication (320 W/15 min)	Food, cosmetics, and pharmaceutical applications
(Himanath et al., 2021)	Water (93 wt%); lecithin (2 wt%); lycopene (1 wt%); VCO (4 wt%)	VCO-based nanoemulsion	Ultrasonication (30 min; 20 kHz; 600 W/cm^2^)	Beverages and food
(Syapitri et al., 2022)	Tween 80 (17.3 wt%); VCO (3 wt%); PEG 400 (8.7 wt%); water (70 wt%); ethanolic black cumin oleoresin extract (1 wt%)	VCO-based nanoemulsion	Homogenization (30,000 rpm/30 min)	Lycopene delivery in yogurt; enhances antioxidant properties
(Khor et al., 2014)	Modified starch (1.3 wt%); stevia (1 wt%); gum arabic (1.3 wt%); VCO (9.4 wt%); xanthan gum (1 wt%); potassium sorbate (0.1 wt%); citric acid (0.08 wt%)	O/W emulsion	Homogenization (7,000 rpm/1.5 min)	Food supplement
(Abllah & Shahdan, 2018)	Modified starch (2 × 1 wt%); stevia (2 × 1 wt%); potassium sorbate (2 × 0.1 wt%); gum arabic (2 × 1 wt%); VCO (7.5-10 wt%); xanthan gum (0.8 wt%); citric acid (0.08 wt%)	O/W emulsion	Homogenization (7,000 rpm/1.5 min)	Not applicable
(Tanglao et al., 2019)	VCO (50 v/v%); whey protein isolate; retinyl acetate (0.70 mg)	O/W emulsion	Mixer with hand homogenizer (speed 5)	Vitamin A encapsulation
(Lastri et al., 2020)	Honey (2 wt%); water (18 wt%); VCO (80 wt%); Span 80: Tween 80 (3:2, 0.75 wt%); citric acid (0.02–0.06 g/100 mL)	O/W emulsion	Homogenization (15,000 rpm/4 min)	Not applicable
(Wiyani et al., 2021)	Water (16 wt%); sucrose (4 wt%); citric acid (0.02 wt%); VCO (80 wt%); Span 80: Tween 80 (3:2, 0.75 wt%)	O/W emulsion	Homogenization (15,000 rpm/4 min)	Not applicable
(Hee et al., 2017a)	Soy lecithin (1 wt%); sodium caseinate (4.4 wt%); VCO (11.6 wt%); maltodextrin (13 wt%); water (70 wt%)	Encapsulated VCO powder	Spray drying (supercritical CO₂; 3 mL/min; 40 °C; 16 MPa)	Not applicable
(Hee et al., 2017b)	VCO (30 wt%); microcrystalline cellulose; maltodextrin	Pickering O/W emulsion (encapsulated VCO powder)	Spray drying (5 mL/min; inlet 150 °C ± 3; outlet 70 °C ± 3) + homogenization (14,000 rpm/5 min)	Non-dairy creamer
(Kulandasamy et al., 2023)	Gum Arabic (25 wt%); maltodextrin (75 wt%); VCO (25 wt%); Tween 80	Encapsulated VCO powder	Spray drying (inlet 160 °C; outlet 50-60 °C; flow rate 6 mL/min)	Not applicable
(Mohammed et al., 2020)	Water (60 wt%); VCO (10 wt%); soy lecithin; sodium caseinate; maltodextrin (30 wt%)	Encapsulated VCO powder / emulsion	Homogenization (8,000 rpm / 180 MPa) + freeze-drying + spray-drying	Non-dairy cream powder
(Hariyadi et al., 2022)	Hydrolyzed VCO (5 wt%); sodium alginate (2 wt%); CaCl₂ (2 wt%); PEG (1 wt%); water (q.s.)	VCO microspheres	Syringe extrusion	Not applicable
(Bhattacharjee, 2021)	VCO + green tea extract 3 : 1 (w/w)	Beverage emulsion	Homogenization + encapsulation	Functional beverage / antioxidant drink
(Rosida et al., 2024)	VCO (40%) replacing margarine	Cookie formulation	Baking	Improved texture and functional properties
(Aini et al., 2020)	VCO (15-25%)	Cheese analogue / fat replacer	Emulsification	Dairy alternative

By structuring VCO into oleogels, its functional bioactive components particularly MCFAs and phenolic antioxidants are better protected from oxidative degradation, thereby preserving their health-promoting properties. This enhanced stability not only improves the shelf life and performance of the ingredient but also ensures a more effective delivery of its antioxidant and metabolic benefits in food systems. Such applications exemplify how VCO’s essential biological properties can be strategically utilized in innovative food structures, bridging the gap between its documented health effects and practical functional food applications.

#### 5.2.3 Emulsions based on VCO

Emulsions are gaining recognition as innovative carriers for food supplements, supporting in the delivery and protection of functional compounds like phenolics, lipids, flavonoids, vitamins, and alkaloids derived from natural sources (Widianingrum et al., 2023). The formulation of emulsions using VCO has been introduced in recent studies. Sweeteners like honey, sorbitol, stevia and glucose are commonly added to O/W emulsions, where VCO acts as the oil phase to improve both sweetness and stability (Wiyani et al., 2021). These sweeteners have been shown to increase emulsion viscosity therefore preventing demulsification (Khor et al., 2014). In addition, antimicrobial agents like potassium sorbate and citric acid have been incorporated into emulsions based on VCO to enhance shelf life. Moreover, emulsions formulated with natural polysaccharides, including xanthan gum, gum arabic, stevia, and modified starch, have shown stability for up to three months under 25 °C room temperature conditions (Khor et al., 2014). Such VCO-based emulsions make it easier to incorporate VCO into food products (Khor et al., 2014; Tanglao et al., 2019).

Nano-emulsions, characterized by droplet sizes between 10 and 1000 nm have attracted considerable interest for use in food applications due to their strong kinetic stability (Gani & Benjakul, 2018; Sanjeewani & Sakeena, 2013; Sathya et al., 2017). For example, a nano-emulsion containing ethanolic extract 0.3% w/w of Noni fruit and 4.5% w/w VCO maintained stability for five days (Jose et al., 2022). In a study by Paramita et al. (2022), a nano-emulsion was formulated using alpha-cyclodextrin, VCO, and Tween 80, resulting in a 49.29% (w/w) of solid content. This formulation exhibited no changes in color, odor, or phase separation after three weeks of storage at 4 °C.

Pickering emulsions stabilized by solid colloidal particles instead of low molecular weight surfactants have gained popularity. These particles adsorb at the oil-water interface enhancing emulsion stability by reducing flocculation, Ostwald ripening, and coalescence (Øye et al., 2023). Additionally, a Pickering emulsion of VCO in water, stabilized with microcrystalline cellulose and maltodextrin via homogenization remained stable for three months (Nurhadi et al., 2020; Nurhadi et al., 2021). These emulsions which use VCO as the oil phase exhibit desirable rheological properties, appearance, and stability, and may serve as replacements for unhealthy fats like saturated and trans fats in food products (Øye et al., 2023). Because they are surfactant-free, Pickering emulsions are gaining wider recognition for their applications in food.

Recently, emulsions have been used to produce emulsified films for packaging applications. VCO was integrated into emulsions of sodium alginate, corn starch, and gum arabic, which were subsequently used to produce composite films. These emulsified films exhibited enhanced barrier properties against water and oxygen (Luan et al., 2024). Likewise, the addition of VCO to potato starch films improved their water resistance and mechanical strength. These films based on VCO have also demonstrated the ability to inhibit the growth of *L monocytogenes*, *S aureus*, and *E coli*, rendering them suitable for food packaging applications (Chhikara & Kumar, 2022).

By formulating VCO into emulsions, particularly nano- and Pickering emulsions, its bioactive components such as MCFAs and phenolic antioxidants are protected and efficiently delivered, enhancing their functional and health-promoting effects. These emulsified systems not only improve the stability and bioavailability of VCO’s active compounds but also expand its practical applications in functional foods, vegan alternatives, and active packaging. This demonstrates a direct linkage between VCO’s documented health benefits and its innovative technological applications, fulfilling the “from properties to applications” approach highlighted in this review.

## 6 Conclusions and future perspectives

VCO represents a promising multidisciplinary ingredient bridging nutrition, food science, and health, supported by a growing body of preclinical and clinical evidence. Its distinctive composition, rich in MCFAs (particularly lauric acid) and bioactive compounds such as phenolics and tocopherols, underpins its antioxidant, anti-inflammatory, antimicrobial, and metabolic effects. Mechanistically, VCO modulates oxidative stress, inflammatory signaling pathways (e.g., NF-κB, Nrf2), lipid metabolism, and immune responses. In parallel, its technological versatility enables applications in functional food systems, including fat substitutes, emulsions, oleogels, and encapsulated formulations, effectively translating its biological properties into practical product development. However, evidence regarding its cardiometabolic effects remains inconsistent. While some studies report improvements in HDL-C and metabolic markers, others including meta-analyses indicate increases in LDL-C, raising concerns regarding cardiovascular risk. Given its high saturated fat content, excessive intake of VCO may contribute to adverse lipid profiles, particularly in individuals with obesity or pre-existing cardiovascular diseases. Therefore, VCO should be considered a functional dietary component to be consumed in moderation within balanced diets, rather than a universally beneficial fat.

Future research should prioritize well-designed, long-term, large-scale randomized controlled trials to clarify dose-response relationships, long-term safety, and population-specific effects of VCO, particularly in relation to cardiovascular and metabolic health. Comparative studies with other dietary fats are essential to position VCO within evidence-based nutritional recommendations, while mechanistic investigations using omics approaches and gut microbiota analysis may further elucidate its biological actions. From a food science perspective, continued development of VCO-based delivery systems, including nanoemulsions, oleogels, and encapsulated formulations, will be critical to enhance stability, bioavailability, and functional performance in complex food matrices. Additionally, optimizing formulation strategies in applications such as beverages and baked goods, alongside sustainable processing and byproduct valorization, will strengthen its industrial relevance. Overall, integrating rigorous clinical validation with technological innovation will be essential to establish VCO as a scientifically grounded, safe, and effective functional ingredient.

## CRediT authorship contribution statement

**Imad Khan:** Writing – review & editing, Writing – original draft, Visualization, Formal analysis, Data curation. **Zehua Qiu:** Writing – review & editing. **Jianguo Zhang:** Writing – review & editing. **Rui Li:** Writing – review & editing, Validation, Conceptualization. **Zhiyou Yang:** Writing – review & editing. **Weimin Zhang:** Writing – review & editing. **Shucheng Liu:** Writing – review & editing. **Minmin Tang:** Writing – review & editing, Supervision, Resources, Investigation, Conceptualization. **Qiuyu Xia:** Writing – review & editing, Supervision, Resources, Methodology, Investigation, Formal analysis, Conceptualization.

## Funding

The authors acknowledge the financial support from the National Natural Science Foundation of China (32172252), Central Public-interest Scientific Institution Basal Research Fund (1630012026324; 1630012026329) and the Program for Scientific Research Startup Funds of Guangdong Ocean University (R20077).

## Declaration of competing interest

The authors declare that they have no known competing financial interests or personal relationships that could have appeared to influence the work reported in this paper.

## Data Availability

Data will be made available on request.
